# Viral Agents as Potential Drivers of Diffuse Large B-Cell Lymphoma Tumorigenesis

**DOI:** 10.3390/v14102105

**Published:** 2022-09-22

**Authors:** Esma Bilajac, Lejla Mahmutović, Kenneth Lundstrom, Una Glamočlija, Jasmin Šutković, Abas Sezer, Altijana Hromić-Jahjefendić

**Affiliations:** 1 Department of Genetics and Bioengineering, Faculty of Engineering and Natural Sciences, International University of Sarajevo, Hrasnička cesta 15, 71000 Sarajevo, Bosnia and Herzegovina; 2 PanTherapeutics, Route de Lavaux 49, CH1095 Lutry, Switzerland; 3 Department of Pharmaceutical Biochemistry and Laboratory Diagnostics, University of Sarajevo, Faculty of Pharmacy, Zmaja od Bosne 8, 71 000 Sarajevo, Bosnia and Herzegovina; 4 School of Medicine, University of Mostar, Zrinskog Frankopana 34, 88 000 Mostar, Bosnia and Herzegovina; 5 Scientific-Research Unit, Bosnalijek JSC, Jukićeva 53, 71 000 Sarajevo, Bosnia and Herzegovina

**Keywords:** diffuse large B-cell lymphoma, viral infections, virus-induced tumorigenesis, Epstein–Barr virus, human T-cell leukemia virus type 1, human immunodeficiency virus, simian virus 40, hepatitis viruses

## Abstract

Among numerous causative agents recognized as oncogenic drivers, 13% of total cancer cases occur as a result of viral infections. The intricacy and diversity of carcinogenic processes, however, raise significant concerns about the mechanistic function of viruses in cancer. All tumor-associated viruses have been shown to encode viral oncogenes with a potential for cell transformation and the development of malignancies, including diffuse large B-cell lymphoma (DLBCL). Given the difficulties in identifying single mechanistic explanations, it is necessary to combine ideas from systems biology and viral evolution to comprehend the processes driving viral cancer. The potential for more efficient and acceptable therapies lies in targeted medicines that aim at viral proteins or trigger immune responses to either avoid infection or eliminate infected or cancerous cells. In this review, we aim to describe the role of viral infections and their mechanistic approaches in DLBCL tumorigenesis. To the best of our knowledge, this is the first review summarizing the oncogenic potential of numerous viral agents in DLBCL development.

## 1. Introduction: Diffuse Large B-Cell Lymphoma

Diffuse large B-cell lymphoma (DLBCL) is one of the most common types of non-Hodgkin lymphoma (NHL) [1,2]. The disease accounts for approximately 30–40% of the total number of newly diagnosed B-cell NHL cases in multiple geographical regions, representing significant health problems that affect millions of people worldwide [2,3,4]. DLBCL occurs as a result of malignant B-cell proliferation during different stages of cell development in which cell morphology, immunophenotyping and genetics define a distinct cell of origin (COO) [5,6]. Although DLBCL can be diagnosed in young patients, the median age of diagnosis occurs between the sixth and the seventh decades [7]. Characterized as an aggressive disease, DLBCL is represented by rapidly growing tumor mass or lymph node enlargement in different nodal and extranodal sites. B-cell symptoms observed in approximately 30% of patients involve weight loss, night sweats and fever [1,2,8]. Besides distinct morphology, genetics and molecular pathogenesis, these subtypes respond differently to the standard chemotherapeutic regimen, including R-CHOP (cyclophosphamide, doxorubicin, vincristine, prednisone plus the monoclonal anti-CD20 antibody rituximab) [9], as well as regimens containing targeted therapies [1,8]. 

Gene expression profiling (GEP) is frequently used to distinguish COO, classifying DLBCL into different subgroups: activated B-cell (ABC) (50–60%) and germinal center B-cell (GCB) (40–50%) DLBCL, as well as a small unclassified group [5,10,11]. Derived from the plasmablastic stage, the ABC subtype is characterized by the constitutive activation of nuclear factor kappa B (NF-κB) signaling and the expression of genes characteristic for mature plasma cells [12]. Other pathological signatures contributing to ABC DLBCL include the inactivation of B-lymphocyte-induced maturation protein 1 (BLIMP1) [13,14] and NF-κB negative regulator A20 [15,16,17]. In addition, mutations in the immunoreceptor tyrosine-based activation motif (ITAM) of CD79B associated with antigen-activating chronic B-cell receptor (BCR) signaling [18] are also observed in ABC DLBCL samples. Mutations in the caspase recruitment domain-containing protein 11 (CARD11) and the myeloid differentiation primary response 88 (MYD88) result in the canonical activation of NF-κB signaling, promoting cell proliferation and differentiation [12,14,19]. Other hallmarks contributing to ABC DLBCL pathogenesis involve chromosomal translocations in the *B-cell lymphoma 6* (*BCL6*) gene, the overexpression of interferon regulatory factor 4 (IRF4), the amplification of Bcl-2 protein and other factors [13,15,17,20,21,22,23,24].

In contrast, the central biological feature of GCB DLBCL is gene mutations involved in immune receptor signaling and epigenetic regulation [25]. GCB DLBCL originates from highly proliferative centroblasts in the germinal center and employs a distinct activation mechanism, referred to as phosphatidylinositol 3 kinase (PI3K) signaling [15,26]. One of the most common GCB DLBCL abnormalities is the 3q27 chromosomal translocation found on the *BCL6* locus, as well as the t(14;18) translocation in the *BCL2* gene, resulting in cell survival and the evasion of apoptosis, respectively [27,28]. Other pathological signatures of GCB DLBCL include the deregulation or loss of phosphatase and tensin homologue (PTEN), leading to the constitutive activation of the PI3K/Akt/mTOR signaling pathway [22,23,29]. Moreover, overexpression of the strong oncogene miR-17-92 detected in GCB patient samples results in rapid cell proliferation, angiogenesis, cell survival and the induction of malignant transformation. Gain-of-function mutations affect the coding region of enhancer of zeste homologue 2 (EZH2), a histone lysine N-methyltransferase enzyme, resulting in the increased silencing of multiple tumor suppressor genes, which promotes cell survival and proliferation, also an important hallmark of GCB DLBCL [29,30,31].

Besides different molecular, clinical features and frequently recurring mutations that contribute to DLBCL pathogenesis, environmental factors, including viruses and bacteria, may also be implicated in DLBCL and the development of different cancer types [32,33]. Viruses have been found to contribute to the development of cervical, hepatocellular, head and neck cancer, lymphoproliferative disorders and other cancers through the activation of innate immune responses and intracellular signaling mechanisms controlling cancer cell growth and viral infection [33,34,35,36,37,38]. The data from the GLOBOCAN 2018 study showed that 2.2 million cancer cases in 2018 were attributable to infections [39]. Viruses are among the most important infectious agents responsible for this burden, representing about 13% of all cancer cases, excluding non-melanoma skin cancer [39]. 

The first virus linked to cancer was the Rous sarcoma virus (RSV) identified in 1910 as a sarcoma-inducing virus in chickens [40]. Several other viruses implicated in tumor development include human papillomaviruses (HPV), Epstein–Barr virus (EBV), hepatitis B (HBV) and hepatitis C virus (HCV), human T-cell lymphotropic virus (HTLV-1), human herpesvirus 8 (HHV-8) or Kaposi’s sarcoma-associated herpesvirus (KSHV), Merkel cell polyomavirus (MCPyV), human cytomegalovirus (HCMV), human immunodeficiency virus (HIV), Simian virus 40 (SV40), BK virus (BKV) and John Cunningham virus (JCV) [38,40,41,42].

Viral proteins encoded by oncoviruses cause deregulation of the cell cycle, inhibition of apoptosis and inactivation of tumor suppressor proteins [42]. Additionally, the majority of DNA and RNA viruses integrate their viral genome into the host cell, resulting in different cellular transformations and chromosomal rearrangements that further contribute to the development of cancer [42,43].

Various reports suggest an association of viral infections with DLBCL, including HIV, EBV, HTLV-1, HBV, HCV and SV40 infections [33,44,45,46,47,48,49,50], while HHV-8 and HPV association with DLBCL is observed only in rare cases [51,52].

Insights into viral etiology in cancer are important for the assessment of risk and prevention, as well as the identification of potential therapeutic targets for drug and vaccine development [53].

## 2. Viruses in DLBCL

**Figure 1:**
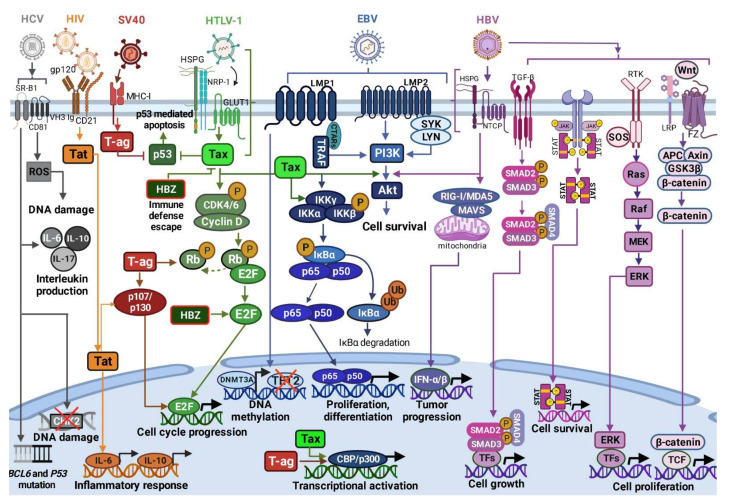
Mechanism of B-/T-cell infection by different viral agents and their effect on the intracellular signaling pathways involved in DLBCL tumorigenesis. The figure was created using BioRender (https://app.biorender.com/ (accessed on 21 July 2022)).

Viral infections associated with DLBCL pathogenesis deregulate the intracellular signaling pathways involved in cell survival, proliferation, differentiation, apoptosis, cell cycle progression and other functions. Figure 1 summarizes the mechanisms of B-/T-cell infection by EBV, HTLV-1, HIV, SV40, HBV and HBC, followed by interference with protein targets as key players regulating distinct cellular processes [42,48,54,55,56,57,58,59,60,61,62,63,64,65,66,67,68,69,70].

### 2.1. Epstein–Barr Virus

Epstein–Barr virus (EBV)-positive DLBCL, not otherwise specified (NOS), is characterized by a distinct clinical presentation and a frequency rate of 2.5–14.0%. This disease is mainly diagnosed in male patients over 50 years of East Asian origin. DLBCL patients with a high viral DNA load and EBV-encoded RNA (EBER) are usually associated with worse prognoses and have inferior responses to the R-CHOP regimen compared to those with EBV-negative DLBCL [45]. 

EBV or human gammaherpesvirus 4 (HHV-4) is an oncogenic virus belonging to the *Gammaherpesvirinae* subfamily which infects more than 90% of humans, while annually 1% of global cancers are associated with EBV infection. Viral exposure usually occurs in childhood and is transmitted through oral secretion and blood transfusion [71,72,73]. The infection occurs in two phases, including primary infection, in which the EBV infects epithelial cells or B-lymphocytes of the oropharynx. The disease is mostly asymptomatic in children, while infection among adolescents results in infectious mononucleosis [45,74]. 

Upon infection, viral replication is initiated to produce virions that result in cell lysis. Lytic EBV replication occurs in host epithelial cells, while in the latent infection phase the virus infects B-cells [45]. Although natural killer cells and cytotoxic T-cells successively eliminate infected B-cells, the downregulation of antigen expression allows certain cells to escape destruction. These cells can then pass through the germinal center and exit as EBV-infected memory cells. The expression of latent genes in B-cells might result in B-cell lymphomas due to cell transformation. Similarly, the EBV is capable of switching from lytic stage genes in epithelial cells to the expression of latency genes, resulting in transformation and constitutive cell proliferation, which leads to cancer development [45,71,72,73,74,75,76].

The genome of EBV is composed of double-stranded DNA with more than 85 open reading frames (ORFs) (Table 1) [77]. During latent phases of infection, different viral genes are expressed with diverse biological properties. Latency III is observed in approximately 7–28% of EBV-DLBCL positive patients, in which six EBV nuclear antigens (EBNAs) are expressed; EBNA 1, 2, 3A, 3B, 3C and LP. In latency III, latent membrane proteins (LMP) (LMP1, 2A and 2B) as well as EBER are expressed. Latency II status is characteristic for the majority of EBV-positive DLBCL patients with EBNA1, LMP1/2A and EBER expression. Additionally, during the latency I phase, which is mostly present in Burkitt’s lymphoma patients, the expression of EBNA1 and EBER is detected [78,79,80]. According to Crombie and LaCasce (2019), LMP1 is expressed in approximately two-thirds of DLBCL patients corresponding to latency phase II, while the rest are characterized by EBNA2 expression, which denotes latency type III [6].

Interestingly, the constitutive activation of NF-κB signaling, regardless of DLBCL subtype, is observed in EBV-positive DLBCL. The main reason for the activation of NF-κB is the viral expression of LMP1 and LMP2A, which mimics the B-cell receptor and CD40 signaling pathways. LMP1 as an integral membrane protein activates NF-κB through TRAF activation. As a result, PI3K and Akt are also activated, leading to cancer cell survival and growth. EBV-positive DLBCL bear several pathological features, including atypical lymphocytes of middle to large sizes with nodular proliferation [54,55,56,57]. Therefore, two distinct patterns of EBV-positive DLBCL are recognized, one characterized by the polymorphic, dominant pattern with an increased number of plasma cells, histiocytes, epithelioid cells and small lymphocytes. The other, the monomorphic subtype, is observed in 2–23% of cases with transformed B-cells of large sizes [6,54,79,81]. 

The main signature of EBV-positive DLBCL is the host immune response, while the genetic profiles of EBV-positive and -negative DLBCL patients differ in several key points [82]. Importantly, DNMT3A is frequently found in EBV-positive DLBCL, while mutations in *CD79B*, *CARD11* and *MYD88* as well as the chromatin remodeling gene *EZH2* are not detected in these patients [83,84,85]. One of the interesting features of EBV-positive DLBCL is that 63–100% of ABC DLBCL patients are positive for IRF/MUM1, while being negative for CD10 and BCL6 [6,45]. Besides the immunohistochemical profile screening of B-cell markers, the most important diagnostic test with great sensitivity is in situ hybridization for EBER [86].

EBV-positive DLBCL patients with 2-year median survival rates have worse prognoses than patients with EBV-negative tumors. Besides the standard treatment regimen, including R-CHOP, there is no specifically accepted treatment for EBV-positive DLBCL patients [87,88]. In a retrospective study (*n* = 1696 DLBCL-NOS) that included 70 cases of EBV-positive DLBCL, [89] reported latency type II (88%) and III (12%) phases with non-GCB phenotypes among all patients. Patients were treated with chemotherapy (22%), immunochemotherapy (59%) and palliative care (19%). The 5-year progression-free survival (PFS) and overall survival (OS) rates were 52.7% and 54.8%, respectively, after a 48-month follow-up period. EBV-positivity in DLBCL patients was correlated with poorer survival rates, especially in older patients compared to patients with EBV-negative DLBCL statuses [89].

Currently, there is no conventional treatment for EBV-positive DLBCL, and the choices are largely consistent with existing treatments for de novo DLBCL [90]. 

During latency, EBV inhibits the synthesis of highly immunogenic proteins and produces lytic proteins that hinder antigen processing by infected cells, as well as viral cytokines that damage the immune system [91]. The efficacy of adoptive T-cell treatment with specific EBV cytotoxic T-cells (CTLs) in patients with EBV-related post-transplant lymphoproliferative disease (PTLD) was investigated and was found to be an effective and safe therapy [92]. The infusion of EBV CTLs was beneficial as therapy and as a prophylactic approach in patients undergoing transplantation [93]; nevertheless, the extended production time may be too long for patients with aggressive malignancy.

The expression of program death ligand 1 (PD-L1) is a potential biomarker with therapeutic relevance in EBV+ DLBCL. The upregulation of PD-L1 is an immune evasion strategy in numerous malignancies, inactivating anti-tumor T-cell responses [94]. A study of younger EBV+ DLBCL patients found higher levels of PD-L1 in tumor cells as well as non-malignant histiocytes [80]. Targeting the PD-L1/PD-1 pathway might be a promising treatment strategy for EBV+ DLBCL.

**Table 1:** Oncogenic viruses and their modes of action in DLBCL development.

Virus/Parameter	Genome	Mechanism(s) of Infection	Infection Outcomes in DLBCL	Therapeutic Approach in DLBCL
**EBV**	ds DNA [77]	Latency III Expression of nuclear antigens EBNA 1, 2, 3A, 3B, 3C, and LP. Latent membrane proteins (LMP) 1, 2A and 2B and EBER expression. Latency II: Expression of EBNA1, LMP1/2A and EBER. Latency I: Expression of EBNA1 and EBER [60,61,62].	LMP1 and LMP2A mimic the B-cell receptor and CD40 signaling pathways, constitutively activating NF-κB signaling [6,54,79,81].	R-CHOP treatment [87,88]
**HTLV-1**	ss RNA [59,60]	Cell-to-cell manner through GLUT1 receptor. Infected cells form virological synapses with uninfected cells [95,96]	Inhibition of p53; activation of PI3K/Akt signaling [62,63,97]; constitutive activation of NF-κB signaling [64,65]; Stimulation of *CCND2* gene expression and phosphorylation of Rb protein [97,98,99]	CHOP and R-CHOP treatment [48,90]
**HIV**	ss linear RNA [66]	Direct mechanisms: Interaction with B-lymphocyte surface molecules: viral glycoprotein gp120, membrane immunoglobulins, C-type lectin receptors [100,101]; CD1 and CD21. Indirect mechanisms: Regulation of cytokine secretion deregulating B-cell differentiation and activation, leading to DNA modifications [102] Low CD21 levels and low B-cell response to antigenic stimuli; Induction of B-cell hyperactivation by increasing expression of activation-related molecules [103].	Direct cell cycle control by Tat through Rb2/p130 protein interaction [66,67]	Antiretroviral therapy (HAART) [46,104]; R-CHOP and R-EPOCH [105]
**SV40**	ds circular DNA [106]	Viral entry/endocytosis by interaction with major histocompatibility complex class I (MHC-I) molecules [107,108].	T-ag-mediated inhibition of tumor suppressors p-Rb, p53, p107 and p130 [68,69,109]; T-ag- induced dissociation of E2F from p-RB [68,69,70,106,110]; Induction of cell growth, cell transformation and resistance to apoptosis by increased levels of the CBP/p300 protein [69,111,112].	-
**HBV**	ds circular DNA [113,114]	Cell membrane interaction with glypican 5 or heparan sulfate proteoglycans, following clathrin-mediated endocytosis coordinated by EGFR into the endosomal network. HBV transition into the nucleus [115,116]	Activation of RIG-I, NF-κB, Wnt/β-catenin, TGF-β, GAS-STING, PI3K/AKT, JAK/STAT, RAS/MEK/ERK signaling pathways; Increased production IFN-α/-β, IL-1 and IL-6; Inhibition of TLR signaling, reduced production of TNF-α [117].	Rituximab [118]
**HCV**	ss RNA [119]	Viral uptake and internalization by claudin-1, CD81, and SR-BI receptors [120]	*BCL6* and *p53* gene mutations [121]; Increased IL-6, IL-10, IL-17, and TGF-β production [122,123,124]; NS3 protein overexpression induces ROS leading to DNA damage [122,125,126]	Rituximab [127]; AVT [128]

In summary, EBV-positive DLBCL is a heterogenous entity with a distinct histological and clinical presentation, as well as an aggressive manifestation in comparison to EBV-negative DLBCL [45,89]. Currently, numerous clinical trials are evaluating the activity of different drugs in EBV-positive DLBCL cases, including sintilimab, an anti-programmed cell death protein 1 (anti-PD-1) antibody in combination with R-CHOP (NCT: 04181489), a combination of zanubrutinib (Bruton’s tyrosine kinase inhibitor) and anti-PD-1 monoclonal antibody tislelizumab (NCT: 04705129), and a late-phase clinical trial of nanatinostat (class I selective histone deacetylase inhibitor) in combination with the antiviral drug valganciclovir (Nana-val) (NCT: 05011058). Information related to other recruiting clinical trials is available at https://clinicaltrials.gov/ (accessed on 20 July 2022).

Considering the complex molecular landscape of EBV-positive DLBCL with poorer survival rates for the standard R-CHOP treatment regimen, there is an urgent need for the development of specific and more effective treatment strategies for EBV-positive cases.

### 2.2. Human T-Cell Leukemia Virus Type 1

Human T-cell leukemia virus type 1 (HTLV-1), which belongs to deltaretroviruses, is a causative agent of an aggressive T-cell malignancy known as adult T-cell leukemia (ATL) [58,59]. It was the first retrovirus to be associated with development of malignancy [97]. Approximately 4–7% of HTLV-1-infected individuals develop ATL, which has been correlated with poor prognoses as a result of therapeutic resistance and immunosuppression [129,130]. 

HTLV-1 has the ability to infect different cell types, such as B-cells and T-cells, as well as synovial cells. The viral transmission occurs in a cell-to-cell manner through the glucose transporter type 1 receptor (GLUT1), in which the infected cells form virological synapses with the uninfected cells [95,96]. Additionally, the virus is transmitted in a vertical manner during childbirth and breastfeeding, as well as sexual contact and blood transfusions [131]. ATL, resulting from an HTLV-1 infection, is characterized by a long period of latency; the disease appears up to 30 years after primary infection. The highest disease prevalence is observed in Central Africa, Japan and the Caribbean, occurring more commonly in male than female populations with a median age of 40–50 years [130,132].

HTLV-1 is a single-stranded RNA virus with a linear genome that generates double stranded DNA and integrates it into the host genome at different chromosomal integration sites (Table 1). HTLV-1 contains a unique pX region at the 3′ end, encoding Tax, HTLV-1 bZIP factor (HBZ) and Rex. Additionally, the genome codes for structural proteins and enzymes, including pol, env and gag [59,60]. The Tax and Hbz gene products are both linked to oncogenesis and pathogenic transformation in ATL. The transactivator Tax is a 40-kDa protein interacting with various transcription factors, cyclic AMP-responsive element binding protein (CREB) and p300/CBP co-activator, resulting in viral transcription and the deregulation of gene expression, which leads to a resistance to apoptosis and cell transformation [48,58,60,61]. Through the inhibition of the p53 N-terminal activation domain, the Tax protein has the ability to inactivate its transactivation function, which induces G1 cell cycle arrest due to a DNA damage response. In order to convert the normal cellular function into ATL, HTLV-1 uses Tax to evade apoptosis through the activation of PI3K/Akt signaling by direct binding to PI3K. This results in the activation of downstream transcription factors, such as activator protein 1 (AP1), which promote cell survival and proliferation [62,63,97]. 

Moreover, numerous studies have shown that HTLV-1-infected cells are characterized by the constitutive activation of the NF-κB signaling pathway which promotes cell differentiation. The Tax protein binds IKKγ in the cytoplasm, which results in phosphorylation of IKKα and IKKβ. The activation of the complex further phosphorylates IkBα which is degraded by proteasome-mediated degradation, in which the NF-κB transcription factor translocates into the nucleus, promoting the transcription of numerous cell proliferation genes [64,65]. Tax stimulates the expression of numerous other genes, such as the *CCND2* gene encoding cyclin D1 that regulates the G1-S-phase transition. Furthermore, the activation of cyclin-dependent kinases (CDK4/6) by the direct binding of Tax results in the phosphorylation of retinoblastoma (Rb) protein releasing transcription factor E2F1, which accelerates the transition from the G1 to S phases in the cell cycle. In contrast, through direct binding to E-box binding proteins, Tax induces the transcriptional repression of CDK inhibitors INK4C, INK4D and KIP1 [97,98,99]. Tax expression is not an essential factor for ATL development, since 60% of ATL cases lack Tax expression [133]. On the contrary, numerous studies indicated that HBZ is consistently expressed in ATL cases. This protein suppresses Tax and other major HTLV-1 genes, allowing the virus to escape from the host immune defense, thus further promoting infection [97,133,134]. Although HBZ acts as a negative regulator of Tax through the prevention of CREB-2 recruitment to cAMP response element sites and TRE, HBZ still targets other transcription factors, such as Jun-D proto-oncogene, altering cellular proliferation [60]. HBZ was also found to upregulate the transcription of the E2F1 gene, promoting cell cycle progression and proliferation [97].

To the best of our knowledge, the number of reports suggesting association between DLBCL and HTLV-1 is limited. A retrospectively analyzed relationship between head and neck B-cell NHL (*n* = 198) treated with chemotherapy and/or radiotherapy and HTLV-1 infection was reported [135]. Out of 21 HTLV-1 seropositive and 177 seronegative cases, the 5-year overall survival (OS) was longer in patients negative to HTLV-1 (78%) compared to those with seropositive cases (49%). In addition, among the DLBCL patient samples (*n* = 26), 12 cases were seropositive, while the rest showed seronegative HTLV-1 statuses. The results demonstrated that B-NHL patients infected with HTLV-1 have a poorer prognoses compared to non-infected individuals. However, HTLV-1 status was not a significant indicator of the OS rate in DLBCL patients and therefore was not linked to DLBCL lymphomagenesis [135]. 

Ref. [48] reported seven de novo DLBCL patient cases seropositive for HTLV-1. Among three patients treated with R-CHOP and CHOP regimens, the 2-year OS was 71%. Interestingly, three patients were characterized by the presence of EBER in tumor cells, suggesting a potential incidence of EBV in HTLV-1-positive DLBCL patients, which could insinuate a lymphomagenetic interaction between the two viruses. Despite the hypothesis indicating a pathogenic link between EBV and HTLV-1, whose interaction might promote the deregulation of T- and B-cells, the small sample size is still a great limitation for the generation of conclusive data. Therefore, further research is required for a better understanding of EBV and HTLV-1 interaction [48,90]. 

In one of the largest case series, the correlation of DLBCL patients (*n* = 192) with HTLV-1 status was evaluated. In the study, seventy cases (37%) were positive for HTLV-1 infection, while the rest were HTLV-1 negative. The report suggests no significant impact on the 5-year OS and event-free survival (EFS) rates in DLBCL patients treated with the standard therapeutic regimen in regard to the presence of HTLV-1. In this study, the 5-year OS rates in HTLV-1 positive and HTLV-1 negative patients were 40% and 42%, respectively, while event-free survival (EFS) rates were 33% in HTLV-1 positive and 32% in HTLV-1 negative patients. Even though further studies are required to evaluate the potential impact of HTLV-1 infection in DLBCL, so far no significant correlation of HTLV-1 infection as a risk factor for disease progression and higher mortality rates was observed in DLBCL patients [129,136].

### 2.3. Human Immunodeficiency Virus

Human immunodeficiency virus (HIV) carrying a single-stranded RNA genome belongs to the lentiviruses of the retrovirus family and possesses the capability to integrate into host chromosomal DNA using a DNA intermediate [66]. 

HIV gene products, specifically the envelope protein gp120, the accessory protein negative factor Nef, the matrix protein p17, the transactivator of transcription Tat and the reverse transcriptase RT, are considered as potentially oncogenic [66,67,137]. The Tat protein specifically transactivates cellular genes, such as *IL-6* and *IL-10,* and can directly control the cell cycle by interacting with the regulatory protein Rb2/p130. This role of the Tat protein represents a significant factor in the pathogenesis of HIV [66,67]. All HIV proteins can be released from the infected or expressing cells and induce oxidative stress. These characteristics underlie their ability to infect epithelial cells, triggering malignant transformation, and to boost the tumorigenic features of transformed or cancer cells [137]. HIV proteins act alone or are accompanied by other onco-proteins produced by human HBV, HBC and HPV, presenting a carcinogenic risk [137]. Additionally, EBV has been linked to HIV since it is present in 40–50% of HIV-associated lymphomas [138].

In vitro evidence indicates that HIV has transformational properties in B-cells and promotes lymphomagenesis via direct and indirect mechanisms [103]. Direct mechanisms of HIV infection include interactions with surface molecules on B lymphocytes, including: (i) viral glycoprotein gp120 interaction with membrane immunoglobulins from the variable heavy chain 3 (VH3) family; (ii) interaction with C-type lectin receptors on B lymphocytes [100,101]; (iii) binding to CD1 in B cells; and (iv) CD21-dependent interactions [102]. Indirect mechanisms refer to the regulation of cytokine secretion, which deregulates B-cell differentiation and activation, leading to DNA modifications. HIV elevates IL-6 and IL-10 secretion, and changes TNF-α release by monocytes encountered by viral glycoproteins [139].

Although T-CD4 lymphocyte depletion represents a hallmark of HIV infection, virus-related effects are also present in the B-cell compartment. Major B-cell alterations upon HIV infection are low CD21 levels, restructuring B cells to become less responsive to antigenic stimuli and shifting them to pro-plasmacytic pathways. Additionally, HIV induces B-cell hyperactivation by changing surface markers, specifically increasing the expression of activation-related molecules [103]. Studies have shown that HIV patients have elevated base levels of CD86 and CD80, but their B-cells could not up-regulate the expression of those markers after stimulation [140]. Likewise, B-cells from HIV-infected patients had higher CD38, but were also more prone to apoptosis due to an increased number of pro-apoptotic CD95 molecules [141]. These data help define the contradiction of the hyperactivation phenotype: even though B-cells were active at resting state, they responded weakly to adequate stimuli, which resulted in impaired humoral responses [103].

HIV infections contribute to an increased incidence of aggressive lymphoma, most commonly DLBCL and Burkitt’s lymphoma [142]. HIV-caused lymphomagenesis is significantly related to immunosuppression prompted by HIV infection. Approximately 80% of NHL cases have progressive systemic disease and excessive levels of lactate dehydrogenase [66,103]. The bigger risk for lymphoma amid infected individuals is related to several factors: the duration and level of immunosuppression, the production of cytokines involved in B-cell proliferation, and the infections with other oncogenic herpesviruses [138].

When compared with the general population, the risk of DLBCL is increased 60–200-fold in patients with HIV infection [66,103]. HIV-associated DLBCL rises in lymph nodes, but tends to involve any extranodal site, most commonly in the brain, gastrointestinal tract, liver and bone marrow [66].

However, since 1996, highly effective antiretroviral therapy (HAART) has significantly reduced this risk [46,104] due to the improved CD4 counts [66,103]. Moreover, HAART presented other effects on the epidemiologic characteristics of HIV-related lymphoma. HIV-associated DLBCL represents a clinically and pathologically heterogeneous category with a similar morphological spectrum to non-HIV-associated DLBCL, but with differences in the basic pathogenesis [66]. The incidence of highly aggressive immunoblastic DLBCL was reduced from 38% of HIV-associated non-Hodgkin lymphoma cases before to 19% after HAART treatment became available [143]. In contrast, the incidence of centroblastic DLBCL increased from 21% to 44% of cases [66].

Furthermore, immunological recovery after HAART allows for the administration of full-dose chemotherapy without the danger of deadly infections, resulting in better results [144], although standard immunochemotherapy with R-CHOP or etoposide, prednisone, vincristine, cyclophosphamide and doxorubicin (R-EPOCH) has shown good outcomes with limited toxicity [105]. Besson et al. investigated the clinical impact of tumor pathobiology in HIV-associated DLBCL patients treated with an R-CHOP regimen in the multicenter, prospective ANRS CO16 LYMPHOVIR cohort trial. The data revealed that in the HAART era, the biological pathogenesis of HIV-infected DLBCL became increasingly comparable to that of non-HIV infected DLBCL. The enhancement in immunological function linked with HAART can explain this trend. Their findings revealed a strong independent and unfavorable prognostic impact of BCL2 expression in PLHIV with DLBCL (HR: 4·48 95% CI: [1 · 00; 20 · 10], *p* = 0 · 05). These findings are similar to those of other trials of HIV-uninfected patients with DLBCL who were treated with a rituximab- and doxorubicin-based regimen [46,145]. Previous clinical investigations of the LYMPHOVIR cohort revealed that the PFS in PLHIV with DLBCL was not different from that of their HIV-negative counterparts, and that only lymphoma-specific clinical variables predicted survival in this scenario [46]. According to the findings of [146], tumor histogenesis is an independent predictor of lymphoma-specific survival in PLHIV with DLBCL in the cART era. These findings may encourage the use of BCL2-targeted drugs in future prospective investigations of HIV-infected DLBCL patients [146]. 

### 2.4. Simian Virus 40

Simian virus 40 (SV40) was first recognized as a polio vaccine contaminant prepared in rhesus macaque kidney cells, in which immunization with the mentioned vaccines represented a major SV40 exposure source. In the 1960s, SV40 was characterized as tumorigenic, transforming numerous cell types in newborn hamster models [147,148,149]. SV40 is a small, non-enveloped DNA virus belonging to the *Polyomaviridae* family [106]. The double-stranded, circular genome of SV40 is divided into two regions, including early and late regions. In the early region, large and small T antigens (T-Ags) are encoded, while the late region codes for viral capsid proteins (VPs), including VP1-3 [68,106]. T-ag is the main transforming protein required for the initiation of viral DNA synthesis, as well as the stimulation of host cell entry into the S phase of the cell cycle. With the ability to transform host cells through interaction with tumor suppressor proteins, SV40 is accepted as a potential oncogenic virus correlated to neoplastic transformations in the brain, bone tumors and NHLs, as well as malignant mesothelioma [69,106,150].

SV40 possesses the ability to infect numerous cell types, since major histocompatibility complex class I (MHC-I) molecules represent receptors required for viral entry or endocytosis into the host cell [107,108]. Following cell infection, large and small T-ags produced during viral replication bind and therefore block the activity of various tumor suppressor proteins, such as p-Rb, p53, p107, as well as p130 [68,69,109]. Upon p53 binding, the T-ag protein abolishes its function, which results in the accumulation of genetic mutations, leading to tumorigenesis. Additionally, T-ag induces the dissociation of the E2F transcription factor from p-Rb, thus resulting in gene transcription, which regulates cell growth [68,69,70,106,110]. Tag-induced cell growth is also observed through the T-ag/p53 activation of insulin growth factor 1 (IGF-1) and association with other proteins, including Hsc70, Bub1, Nbs-1, Cul7 and TEF-1. Aside from cell growth, T-ag interferes with the induction of cell transformation and resistance to apoptosis due to increased levels of the CBP/p300 protein [69,111,112]. Considering the ability of T-Ag to inhibit function of p53, whose activity is down-regulated in DLBCL, this mechanism might represent one of the potential steps for induction of DLBCL in patients with SV40 infection [151,152].

SV40 has so far been considered as an important oncovirus involved in the progression of NHL. In a study involving serum samples of NHL patients (*n* = 89; *n* = 31 DLBCL) with a median age of 57 years, 40% of the patients were positive for the VP antigen compared to a control group with a similar median age (*n* = 130). In another group (*n* = 61; *n* = 2 DLBCL) with a median age of 68 years, the prevalence of SV40 B and C peptides in the samples was 43% compared to the control group (*n* = 83) with an SV40 prevalence of 16% and 14%, respectively. Therefore, these data indicated a significant association between NHL and SV40 infection [149].

Similar results were observed in a study in which SV40 T-ag sequences were detected in 42% of NHL patients (*n* = 64/154). Interestingly, non-malignant lymphoid samples (*n* = 186) and cancer cases of different origin used as a control (*n* = 54) showed no expression of T-ag. Among tested HIV-infected as well as uninfected DLBCL patients (*n* = 98), 44 and 28 cases were DNA positive for SV40 and EBV, respectively. Compared to follicular, Burkitt’s and other lymphoma types, the greatest prevalence of SV40 was observed in DLBCL patients. These findings suggest that SV40 might promote a more successful transformational potential in mature B-cells, which results in lymphomagenesis [153].

Another study evaluating the association of SV40 in patients with different hematological malignancies (*n* = 266) indicated that SV40 DNA-positive sequences were most commonly detected in NHL patients (total *n* = 158, positive *n* = 85) with a prevalence of 53.8% in comparison to the control group (*n* = 34). The highest percentage was observed in Burkitt’s lymphoma patients (*n* = 18), of whom 77.7% showed a SV40 DNA-positive status, as well as in DLBCL patients (*n* = 51) with 60.7% cases. The results underline the possibility of SV40 as an important cofactor in NHL and leukemia pathogenesis [154]. 

In order to gain insight into SV40 tumor causality, the relationship between the methylation status of tumor suppressor genes as a common silencing mechanism and the presence of SV40 DNA sequences was evaluated in DLBCL (*n* = 108) and non-tumor samples (*n* = 60). The results showed that 56% of DLBCL patients (*n* = 63) were detected to have SV40 DNA compared to the non-tumor samples (6%). Hypermethylation status evaluated through a methylation-specific PCR showed the highest frequency of methylation in *DAPK*, *CDH1* and *SHP1*, and *p16* tumor suppressor genes, with 74%, 70% and 58% positive cases, respectively. Aside from the fact that the hypermethylation of the mentioned tumor suppressor genes was not observed in non-tumor samples, SV40-negative DLBCL samples had significantly lower hypermethylation statuses. These data indicated that the virus might be characterized by a functional effect in DLBCL [49]. Furthermore, the association between SV40 and germinal center biology, as well as p53 and BCL2 expression, was evaluated in SV40-positive (*n* = 48) and -negative (*n* = 38) DLBCL patients (*n* = 86). The data suggested a more prevalent GCB subtype (81%) and p53 accumulation (69%) in SV40-positive DLBCL patients compared to SV40-negative patients [50]. 

Different reports suggested the presence of SV40 DNA in NHL, mainly DLBCL in 11–62% of cases [49]. However, several studies showed no significant correlation of SV40 and the development of NHL [155,156,157]. Ref. [155] analyzed lymph node biopsies and/or blood samples from 232 patients, of whom 152 had cases of NHL. Among the total number of NHL, including DLBCL (*n* = 29), none of the experimental samples showed positivity for SV40 DNA, even when the data were analyzed using different approaches, suggesting there was no association of SV40 with NHL development [155].

Ref. [156] also showed no interrelationship between SV40 infection and NHL. Although among 74 NHL samples, significant mutuality was found between SV40 infection and NHL, further analyses showed that the association occurred as a result of the cross-reactivity with JCV and BKV. Such observations underlined the importance of the incorporation of biomarkers of cross-reactivity with other viral types that might be interconnected in the NHL etiology [156]. Similarly, a recent study showed no pathological association between SV40 and NHL [157]. In this study, 102 biopsy samples from NHL patients were obtained and analyzed through immunohistochemical staining. The results showed no positivity for SV-T-ag P1 and SV-T-ag P2 using nested PCR and electrophoresis [157]. 

The association of SV40 with NHL development remains controversial. Even though various reports suggest a significant percentage of SV40-positive cases among DLBCL patients, still further research is required to obtain more conclusive data. 

### 2.5. Hepatitis B Virus

Hepatitis B virus (HBV) possesses double-stranded circular DNA and belongs to the *Hepadnaviridae* family [113,114]. The HBV genome is surrounded by a nucleocapsid and three envelope proteins, large (L), middle (M), and small (S), which are essential for hepatocyte attachment [158]. In order to initiate viral cell entry, HBV interacts with glypican 5 or heparan sulfate proteoglycans located on the cell membrane of the hepatocytes [159]. It forms an internalization complex after binding to its receptor, the sodium taurocholate co-transporting polypeptide (NTCP) as well as the epidermal growth factor receptor (EGFR) that acts as a co-receptor. The complex enters the host cell through clathrin-mediated endocytosis, in which EGFR plays an important role in the coordination of HBV into the endosomal network by a mechanism that is still not completely understood [115,160]. However, it is known that viral transport and localization to the late endosome is a crucial factor for the infection. After successful endosomal escape, the HBV nucleocapsid uses the microtubular network for the transition to the nucleus [115,116]. This event is followed by the dissociation at the nuclear pore complex and the relaxation of viral circular DNA, which is converted into a transcription template in the form of covalently closed circular DNA (cccDNA) [115].

HBV is a highly infectious virus infecting approximately 250 million individuals globally on a chronic basis, resulting in millions of fatalities each year [113]. HBV requires several mechanisms to survive, including innate immunity to boost replication [114]. It takes advantage of the growing immune system and the unstable gut microbiota in infants to enhance its persistence [161]. Based on a thorough genomic and transcriptome study from 2018, HBV infections extensively depend on the expression of the *BCL6*, *FOXO1* and *ZFP36L1* genes, as well as the immunoglobulin heavy-chain gene sequences. Accordingly, HBV-related lymphomagenesis occurs via an antigen-independent mechanism, rather than a chronic antigenic simulation model [162]. 

HBV infection is distinct from other viral infections [163]. It was shown that HBV targets innate immune signaling pathways to escape the host’s antiviral responses [164]. Upon host cell entry, HBV infection triggers downstream RIG-I-like, Wnt/β-catenin, TGF-β, GAS-STING signaling pathways, PI3K/Akt, JAK/STAT, RAS/MEK/ERK (Figure 1). Infections also induce the production of antiviral cytokines (IFN-α, IFN-β) and inflammatory cytokines (IL-1, IL-6). In addition, HBV antigens can activate specific cellular signaling pathways to promote HBV replication, which as a result inhibits cell apoptosis. Viral gene expression can regulate HBV replication by the inhibition of Toll-like receptor (TLR) signaling [117], and a reduction in the production of tumor necrosis factor-α (TNF-α), IFN-α and pro-inflammatory cytokines. These modifications lead to the activation of interferon-regulated transcription factor 3 (IRF3), NF-κB and extracellular signal-modulated kinase (ERK)1/2 [117].

The capacity of B-cells to produce HBV-specific antibodies is decreased in patients with DLBCL who are positive for hepatitis B core antibody (HBcAb) and are treated with CD20-targeted therapy. This might potentially stimulate the replication of already infected HBV, increasing the likelihood of serum HBsAg conversion [165]. However, the presence of a considerably greater frequency of HBV infection in patients prior to DLBCL treatment suggests that HBV infection in DLBCL patients is not associated with the use of targeted chemotherapeutic agents. Furthermore, human peripheral blood mononuclear cells (PBMCs) may harbor HBV [166], suggesting that HBV infects antigen-presenting lymphocytes, contributing to lymphoma development [167].Several studies concluded that DLBCL patients with chronic HBV infection had worse prognoses, a higher incidence of hepatic dysfunction during chemotherapy, and distinct clinical features [162,168,169].

Thus, both epidemiological and clinical studies have revealed a link between HBV infection and DLBCL. The etiopathological significance of HBV in lymphomagenesis, on the other hand, is mainly unclear. Ren et al. performed a thorough genetic investigation on DLBCLs from HBV-infected individuals, identifying the different molecular characteristics of these tumors in samples from 275 patients [162]. These findings clearly showed that HBV infection has a role in the development of DLBCL. There was no indication of skewed usage of *IGVH* genes, stereotyped CDR3 sections, or homology of CDR3 areas with anti-HBsAg antibodies when the *IGVH* of a subgroup of HBsAg+ DLBCL in this cohort was examined. The chronic antigenic stimulation paradigm is less preferred for HBV-associated DLBCLs than the standard antigen-driven model. This is backed further by clinical findings showing HBV-associated DLBCLs do not respond to antiviral treatment [83]. HBsAg+ DLBCL should be categorized as a unique subtype of DLBCL based on its different clinical and molecular characteristics.

When compared to other lymphomas, such as T-cell lymphoma and follicular lymphoma, DLBCL showed a greater frequency of HBV infection (about 25–60%) [170]. The function of HBV in DLBCL, especially its link to prognosis, appears significantly more uncertain, with just a few studies providing limited evidence concerning the associations of HBV with DLBCL outcomes, although the results are highly inconsistent [171]. Rituximab has significantly boosted the cure rate of DLBCL compared to the standard regimen. However, while rituximab provided benefits, it significantly increased the risk of viral reactivation and hepatic impairment in DLBCL patients with HBV infection during chemotherapy [118]. In a study, the potential roles of HBV in DLBCL were analyzed and the importance of HBV for the prognosis of DLBCL was assessed in a cohort of 136 patients diagnosed with DLBCL [163]. Before treatment, all patients were subjected to serological tests to exclude combined infection with hepatitis A, hepatitis C and HIV, after which rituximab was suggested as the preferred treatment. Forty patients finally stopped the rituximab treatment in favor of the standard CHOP regimen, mainly due to financial constraints. All recorded patients underwent at least three cycles of the first-line CHOP or R-CHOP regimens [172]. In HBsAg-positive patients, antiviral prophylaxis with lamivudine or entecavir was started at least one week before the commencement of chemotherapy and was stopped 6 months later [172]. Because of the widespread adoption of rituximab’s groundbreaking regimen, measurements became more challenging. The survival rate in the sample was examined independently based on the use of rituximab. The results demonstrated that HBV-infected patients following the R-CHOP regimen had a lower PFS and OS than the HBV-uninfected individuals. However, the condition of HBV infection had no effect on the outcome in patients receiving the standard CHOP regimen [172]. The introduction of rituximab significantly altered the fate of DLBCL patients, resulting in longer remission times and higher survival rates [173]. It was also proven that DLBCL patients with cured HBV infections had a greater risk of viral reactivation after the rituximab-containing treatment than the uninfected individuals [174]. The study may explain why, despite a robust response to rituximab, patients with HBV infection did not benefit from it in terms of long-term survival [172]. Considering the cost burden and the viral reactivation risks of rituximab, treatment alternatives for HBV-infected individuals should be carefully considered.

### 2.6. Hepatitis C Virus

Hepatitis C virus (HCV) is a positive strand enveloped RNA virus from the *Flaviviridae* family, representing a leading cause of liver disease worldwide [119]. The life cycle and spread of HCV are inextricably linked to lipid metabolism. 

The HCV genome is composed of a single-stranded RNA of positive polarity containing a single ORF that encodes a polyprotein cleaved into several structural and non-structural proteins. The ORF is flanked by untranslated regions (UTRs), where the internal ribosome entry site (IRES) is located. The IRES binds the 40S ribosomal subunit and initiates polyprotein translation in a cap-independent manner [175]. The non-structural proteins of HCV include the p7 viroporin, the NS2 protease, the NS3-4A complex harboring protease and NTPase/RNA helicase activities, the NS4B and NS5A proteins and the NS5B RNA-dependent RNA polymerase [176]. Globally, the HCV is estimated to infect 58 million people, with about 1.5 million new annual infections [177]. The life cycle and propagation of HCV are tightly connected to lipid metabolism. To enter the cell, HCV goes through several steps, including viral and cellular factors that trigger virus uptake into hepatocytes. The main receptors controlling the HCV entry are tight junction molecules Claudin-1, tetraspanin CD81 and human scavenger receptor SR-BI [120]. The interaction of the named receptors finally leads to the uptake and cellular internalization of HCV through a process of clathrin-dependent endocytosis. However, sometimes HCV uses glycosaminoglycans and/or low-density receptors on host cells as initial attachment factors or it uses specific features of the cell lipid metabolism to enter the cell [119]. 

Additionally, the involvement of the two HCV envelope glycoproteins, E1 and E2, plays an essential role in virus entry [119]; the envelope glycoprotein E2 is characterized by indirect cell transformation. Following E2-CD81 B-cell receptor interaction, the somatic hypermutation of the Ig gene locus is induced by activation-induced deaminase (AID) [122,123]. AID activation by HCV has been correlated with the induction of mutations in the *beta-catenin*, *BCL6* and *p53* genes in B-cells [121]. BAFF as one of the major factors required for B-cell survival was characterized by up-regulated levels in NHL [178]. An increased number of other cytokines, such as IL-6, IL-10, IL-17, and TGF-β also contribute to the proliferation of B-cells as a result of HCV infection [122,123,124]. Moreover, the overexpression of HCV NS3 protein was found to induce nitric oxide synthase (NOS) and reactive oxygen species (ROS), which might be one of the factors causing DNA mutations and double-strand breaks. This event was also accompanied by the downregulation of cell cycle checkpoint kinase 2 (CHK2) as one of the major regulators of DNA damage [122,125,126].

A great number of meta-analyses [179,180,181,182] found an elevated incidence of BCL in patients with chronic HCV infection compared to that of HCV-negative controls [183]. Moreover, several studies from countries with high rates of HCV infection have found a significant epidemiological link between HCV infection and the development of B-cell NHL [184,185,186]. The significant therapeutic potential of antiviral treatment on HCV-related B-cell proliferation or low-grade B-cell lymphomas suggests that the virus plays a direct role in lymphomagenesis [187,188]. Numerous laboratory experiments confirmed that DLBCL is associated with HCV infections despite its aggressive nature [47]. The first results that correlated HCV with lymphoma were reported more than two decades ago [189]. Later on, several other studies conducted in Sweden, Italy, Spain, North America and Australia concluded there was a strong association between HCV and DLBCL [182,190,191]. A large study conducted in 2011–2015 concluded that among 206 DLBCL patients, 22 (10.7%) were HCV-positive. In comparison to low-grade B-cell lymphomas, the literature on the pathobiology and therapy of patients with DLBCL infected with HCV is relatively limited. Indeed, multiple retrospective investigations have found that individuals with HCV-related DLBCL have distinct features when compared to their HCV-negative peers, indicating that the virus may be involved in the very early stages of lymphomagenesis [192,193,194,195]. 

HCV-positive individuals are typically older, have spleen/liver or extranodal involvement, and have higher lactate dehydrogenase levels. Epidemiological data, on the other hand, are among the clearest arguments in favor of the role of viral infection in the development of DLBCL [47].

Even in the absence of viral entry into the human B-cell, HCV has been demonstrated to protect human B cells against Fas-mediated apoptosis via E2-CD81 interaction [196]. It should also be highlighted that B-cell associated viruses have a higher infectivity than extracellular viruses and may easily infect hepatoma cells. Thus, these viruses can alter B-cell tropism, evade natural immunity, and persist in the infected liver [197].

Various hypothesized models of HCV-induced B-cell transformation have been developed [198]. A hypothetical model of B-cell transformation by HCV, known as direct transformation paradigm has been proposed, in which HCV would infect B cells directly, potentially via CD81-E2 contact, and expresses its oncogenic potential via cellular NOS and NS3/4-mediated alterations in proliferation genes. An indirect transformation hypothesis, on the other hand, would rely on the interaction of E2 and CD81 on the cell surface to cause the production of activation-induced deaminase and somatic hypermutations of immunoglobulin genes and putative proto-oncogenes, resulting in continuous B-cell stimulation. Finally, there is the “hit and run” idea, which is based on virus-induced genetic damage to B-cells mediated by a transiently intracellular virus [199]. 

Given the possible risk of using rituximab in HCV-infected patients, several research studies were undertaken to explore the influence of HCV infection on the survival of DLBCL patients in the rituximab era [127]. Previous research has looked at the impact of antiviral therapy (AVT) on the survival in HCV-infected DLBCL patients. Michot and colleagues discovered a link between AVT and increased OS in 17 of 45 DLBCL patients [128], whereas a better OS was found in 23 of 581 AVT patients in a univariate study [200]. However, both studies investigated the impact of AVT administered after CT and following DLBCL remission, with IFN serving as the basis of AVT. This method was limited by choosing DLBCL survivors and CT responders before using AVT. Another weakness of this strategy was the confusing impact of IFN therapy, which is a powerful anti-lymphoma drug when paired with rituximab [201]. To account for these variables, the effect of AVT on survival only when administered prior to DLBCL diagnosis was examined. Future studies that include HCV-infected individuals treated with AVT during or after CT with non-antineoplastic drugs may give further information on the oncologic benefit of AVT [127].

### 2.7. Other Viruses

HPV is a non-enveloped single-stranded DNA virus transcribed as a bicistronic or polycistronic form containing two or more OFRs [202]. HPV has been associated with more than 95% of cases of cervical cancer. Moreover, HPV has been linked to NHL, primarily derived from the base of the tongue [203]. Among seven identified NHL cases, the most common histologic subtype was DLBCL [203]. One of the DLBCL cases was identified as HPV DNA-positive and diffusively expressed the P16 protein. In another study, tonsil cancer tissue was histologically classified as tonsillar squamous-cell carcinoma (TSCC) and DLBCL in the light of HPV and EBV prevalence [204]. In TSCC, the HPV infection was significantly higher (30.6%) than in DLBCL (13.8%). In contrast, EBV was significantly higher in DLBCL (44.8%) than in TSCC (19.4%) samples.

HHV8, also called KSHV, is a double-stranded DNA virus with a large number of genes of which many show a high homology to human genes, such as *IL-6*, *cyclin D1* and *BCL2* [205]. In a unique case of sickle cell disease, a 59-year-old female showed the sequential development of two separate hematolymphoid neoplasms, HHV-8 positive DLBCL and chronic myelomonocytic leukemia [206]. In the context of Pyothorax-associated lymphoma (PAL), in two out of three new cases, the EBV genome was detected [207]. However, in the third case, no EBV genome was discovered, instead suggesting the co-operation of HHV-8.

## 3. Conclusions

Both DNA and RNA viruses have been identified as causative agents for various cancers, including lymphomas. Viruses such as EBV, HPV, HBV, HCV, HHV-8 and HTLV-1, HIV and SV40 have been associated with DLBLC. Typically, virus-induced pathogenesis targets intracellular pathways affecting cell survival, proliferation, differentiation, apoptosis, and cell cycle functions. Due to differences in genome structure and the viral life cycle of the oncogenic viruses listed above, their mechanisms of action vary. Some viruses, such as EBV, HBV, HPV, HHV-8 and SV40, can integrate their double-stranded DNA into the host genome, whereas for example HTLV-1 and HIV with a single-stranded RNA genome must first generate double-stranded DNA copies for chromosomal integration. In contrast, although HCV contains a single-stranded RNA genome, it does not possess reverse transcriptase activity and therefore cannot integrate into the host cell genome.

In the context of DLBCL, patients who tested positive for EBV show a more aggressive manifestation and worse prognosis of the disease than in EBV-negative individuals. Several clinical trials are in progress for anti-PD-1 antibody treatment combined with R-CHOP and Bruton’s tyrosine kinase inhibitors. Only a limited association between HTLV-1 infection and DLBCL has been reported so far. It has been documented that the OS was longer in DLBCL patients who tested HTLV-1-negative compared to HTLV-1 seropositive cases. There have also been suggestions of a pathogenic association between EBV and HTLV-1, as HTLV-1-positive DLBCL patients showed the presence of EBER in tumor cells. However, it has been concluded that no significant correlation of HTLV-1 infection as a risk factor for disease progress and higher mortality rates of DLBCL could be established. Interestingly, HIV proteins can alone or together with onco-proteins from HBV, HCV, HPV and EBV enhance the carcinogenic risk; for example, EBV has been detected in 40–50% of HIV-associated lymphomas. Moreover, it has been established that HIV contributes to an increased incidence of DLBCL and Burkitt’s lymphoma. Furthermore, the risk of DLBCL is 60–200 times higher in patients with HIV than in the general population. However, HAART therapy has significantly reduced the incidence of highly aggressive immunoblastic DLBCL from 38% to 19% in HIV-associated non-Hodgkin lymphoma patients after HAART treatment. In the context of SV40, studies have indicated a significant association between NHL and SV40 infection with the greatest prevalence in DLBCL patients compared to follicular and Burkitt’s lymphoma. Moreover, it has been demonstrated that SV40 DNA was detected in 56% of DLBCL patients compared to only 6% in non-tumor samples. The hypermethylation of tumor suppressor genes has also been seen at high levels (58–74%) in samples from DLBCL patients but not in SV40-negative DLBCL samples. However, the association between SV40 and NHL development is not clear despite the identification of a large number of SV40-positive cases among DLBCL patients. 

Related to HBV, it has been demonstrated that DLBCL patients with chronic HBV infection show a poorer prognosis and a higher incidence of hepatic dysfunction when subjected to chemotherapy compared to HBV-negative individuals. An association between HBV infection and DLBCL has been revealed in both epidemiological and clinical studies, indicating the role of HBV infection in DLBCL development. Moreover, clinical evaluations have demonstrated that patients with HBV-associated DLBCLs do not respond well to antiviral treatment. For example, HBV-infected DLBCL patients showed a lower PFS and OS than HBV-negative patients after R-CHOP treatment. Moreover, DLBCL patients cured from HBV infections showed a higher risk of viral reactivation after rituximab treatment, which could explain why the HBV-infected DLBCL patients did not show long-term survival, despite having a robust response to the rituximab treatment. In the case of HCV, epidemiological data indicate an association between HCV infection and the development of B-cell NHL. Several studies have confirmed that DLBCL is associated with HCV infections. For example, a large study concluded that 10.7% of DLBCL patients were HCV-positive. The efficacy of antiviral therapy in HCV-positive DLBCL patients monitoring OS has been conducted in several clinical trials. Although an increase in OS was achieved, the treatment only took place after chemotherapy and DLBCL remission, which had a certain impact on the outcome. Therefore, priority should be given to antiviral treatment prior to DLBCL diagnosis.

Although HPV has been linked to 95% of cases of cervical cancer, an association of HPV and NHL has also been established. In seven identified NHL cases, one of the DLBCL cases was HPV DNA-positive. Moreover, it was also demonstrated in tonsil cancer tissue that HPV infections were more frequent in TSCC than DLBCL, whereas for EBV infections it was the opposite. Finally, in a case study, it was reported that a 59-year-old sickle cell disease patient who developed DLBCL was also HHV-8-positive. In another study, two out of three cases of PAL were EBV-positive, while the third was EBV-negative, potentially suggesting an HHV-8 infection. 

In summary, several DNA and RNA viruses have been detected in DLBCL patients. Due to their oncogenic properties, we have presented data on their mechanisms of action and contribution to malignant development. We have also discussed current therapeutic interventions and tried to elaborate on new approaches for better antiviral therapy. 

## Data Availability

Not applicable.

## References

[1] Padala S.A., Kallam A. (2022). Diffuse Large B Cell Lymphoma.

[2] Li S., Young K.H., Medeiros L.J. (2018). Diffuse Large B-Cell Lymphoma. Pathology.

[3] Sehn L.H., Salles G. (2021). Diffuse Large B-Cell Lymphoma. N. Engl. J. Med..

[4] Wang L., Li L., Young K.H. (2020). New Agents and Regimens for Diffuse Large B Cell Lymphoma. J. Hematol. Oncol..

[5] Harrington F., Greenslade M., Talaulikar D., Corboy G. (2021). Genomic Characterisation of Diffuse Large B-Cell Lymphoma. Pathology.

[6] Crombie J.L., Armand P. (2019). Diffuse Large B-Cell Lymphoma and High-Grade B-Cell Lymphoma. Hematol. Oncol. Clin. N. Am..

[7] Kesavan M., Eyre T.A., Collins G.P. (2019). Front-Line Treatment of High Grade B Cell Non-Hodgkin Lymphoma. Curr. Hematol. Malig. Rep..

[8] Chiappella A., Castellino A., Nicolosi M., Santambrogio E., Vitolo U. (2017). Diffuse Large B-Cell Lymphoma in the Elderly: Standard Treatment and New Perspectives. Expert Rev. Hematol..

[9] Epperla N., Hamadani M. (2017). Hematopoietic Cell Transplantation for Diffuse Large B-Cell and Follicular Lymphoma: Current Controversies and Advances. Hematol. Oncol. Stem Cell Ther..

[10] Cioroianu A.I., Stinga P.I., Sticlaru L., Cioplea M.D., Nichita L., Popp C., Staniceanu F. (2019). Tumor Microenvironment in Diffuse Large B-Cell Lymphoma: Role and Prognosis. Anal. Cell. Pathol..

[11] Alizadeh A.A., Eisen M.B., Davis R.E., Ma C., Lossos I.S., Rosenwald A., Boldrick J.C., Sabet H., Tran T., Yu X. (2000). Distinct Types of Diffuse Large B-Cell Lymphoma Identified by Gene Expression Profiling. Nature.

[12] Lenz G., Davis R.E., Ngo V.N., Lam L., George T.C., Wright G.W., Dave S.S., Zhao H., Xu W., Rosenwald A. (2008). Oncogenic CARD11 Mutations in Human Diffuse Large B Cell Lymphoma. Science.

[13] Pasqualucci L., Compagno M., Houldsworth J., Monti S., Grunn A., Nandula S.V., Aster J.C., Murty V.V., Shipp M.A., Dalla-Favera R. (2006). Inactivation of the PRDM1/BLIMP1 Gene in Diffuse Large B Cell Lymphoma. J. Exp. Med..

[14] Lenz G. (2015). Insights into the Molecular Pathogenesis of Activated B-Cell-like Diffuse Large B-Cell Lymphoma and Its Therapeutic Implications. Cancers.

[15] Staudt L.M. (2010). Oncogenic Activation of NF-KappaB. Cold Spring Harb. Perspect. Biol..

[16] Compagno M., Lim W.K., Grunn A., Nandula S.V., Brahmachary M., Shen Q., Bertoni F., Ponzoni M., Scandurra M., Califano A. (2009). Mutations of Multiple Genes Cause Deregulation of NF-ΚB in Diffuse Large B-Cell Lymphoma. Nature.

[17] Lenz G., Nagel I., Siebert R., Roschke A.V., Sanger W., Wright G.W., Dave S.S., Tan B., Zhao H., Rosenwald A. (2007). Aberrant Immunoglobulin Class Switch Recombination and Switch Translocations in Activated B Cell–like Diffuse Large B Cell Lymphoma. J. Exp. Med..

[18] Davis R.E., Ngo V.N., Lenz G., Tolar P., Young R.M., Romesser P.B., Kohlhammer H., Lamy L., Zhao H., Yang Y. (2010). Chronic Active B-Cell-Receptor Signalling in Diffuse Large B-Cell Lymphoma. Nature.

[19] Ngo V.N., Young R.M., Schmitz R., Jhavar S., Xiao W., Lim K.-H., Kohlhammer H., Xu W., Yang Y., Zhao H. (2011). Oncogenically Active MYD88 Mutations in Human Lymphoma. Nature.

[20] Iqbal J., Greiner T.C., Patel K., Dave B.J., Smith L., Ji J., Wright G., Sanger W.G., Pickering D.L., Jain S. (2007). Distinctive Patterns of BCL6 Molecular Alterations and Their Functional Consequences in Different Subgroups of Diffuse Large B-Cell Lymphoma. Leukemia.

[21] Zhang B., Calado D.P., Wang Z., Fröhler S., Köchert K., Qian Y., Koralov S.B., Schmidt-Supprian M., Sasaki Y., Unitt C. (2015). An Oncogenic Role for Alternative NF-ΚB Signaling in DLBCL Revealed upon Deregulated BCL6 Expression. Cell Rep..

[22] Frick M., Dörken B., Lenz G. (2011). The Molecular Biology of Diffuse Large B-Cell Lymphoma. Ther. Adv. Hematol..

[23] Lenz G., Wright G.W., Emre N.C.T., Kohlhammer H., Dave S.S., Davis R.E., Carty S., Lam L.T., Shaffer A.L., Xiao W. (2008). Molecular Subtypes of Diffuse Large B-Cell Lymphoma Arise by Distinct Genetic Pathways. Proc. Natl. Acad. Sci. USA.

[24] Bea S. (2005). Diffuse Large B-Cell Lymphoma Subgroups Have Distinct Genetic Profiles That Influence Tumor Biology and Improve Gene-Expression-Based Survival Prediction. Blood.

[25] Weber A.N.R., Cardona Gloria Y., Çınar Ö., Reinhardt H.C., Pezzutto A., Wolz O.-O. (2018). Oncogenic MYD88 Mutations in Lymphoma: Novel Insights and Therapeutic Possibilities. Cancer Immunol. Immunother..

[26] Basso K., Klein U., Niu H., Stolovitzky G.A., Tu Y., Califano A., Cattoretti G., Dalla-Favera R. (2004). Tracking CD40 Signaling during Germinal Center Development. Blood.

[27] Lossos I.S. (2005). Molecular Pathogenesis of Diffuse Large B-Cell Lymphoma. JCO.

[28] Rosenwald A., Wright G., Chan W.C., Connors J.M., Campo E., Fisher R.I., Gascoyne R.D., Muller-Hermelink H.K., Smeland E.B., Giltnane J.M. (2002). The Use of Molecular Profiling to Predict Survival after Chemotherapy for Diffuse Large-B-Cell Lymphoma. N. Engl. J. Med..

[29] Frontzek F., Lenz G. (2019). Novel Insights into the Pathogenesis of Molecular Subtypes of Diffuse Large B-Cell Lymphoma and Their Clinical Implications. Expert Rev. Clin. Pharmacol..

[30] Dal Bo M., Bomben R., Hernández L., Gattei V. (2015). The MYC/MiR-17-92 Axis in Lymphoproliferative Disorders: A Common Pathway with Therapeutic Potential. Oncotarget.

[31] Béguelin W., Popovic R., Teater M., Jiang Y., Bunting K.L., Rosen M., Shen H., Yang S.N., Wang L., Ezponda T. (2013). EZH2 Is Required for Germinal Center Formation and Somatic EZH2 Mutations Promote Lymphoid Transformation. Cancer Cell.

[32] Liu Y., Barta S.K. (2019). Diffuse Large B-cell Lymphoma: 2019 Update on Diagnosis, Risk Stratification, and Treatment. Am. J. Hematol..

[33] Högfeldt T., Jaing C., Loughlin K.M., Thissen J., Gardner S., Bahnassy A.A., Gharizadeh B., Lundahl J., Österborg A., Porwit A. (2016). Differential Expression of Viral Agents in Lymphoma Tissues of Patients with ABC Diffuse Large B-Cell Lymphoma from High and Low Endemic Infectious Disease Regions. Oncol. Lett..

[34] Jarrett R.F. (2006). Viruses and Lymphoma/Leukaemia. J. Pathol..

[35] Moore P.S., Chang Y. (2010). Why Do Viruses Cause Cancer? Highlights of the First Century of Human Tumour Virology. Nat. Rev. Cancer.

[36] Ringelhan M., McKeating J.A., Protzer U. (2017). Viral Hepatitis and Liver Cancer. Philos. Trans. R. Soc. Lond. B Biol. Sci..

[37] Wardak S. (2016). Human Papillomavirus (HPV) and Cervical Cancer. Med. Dosw. Mikrobiol..

[38] Mahmutović L., Bilajac E., Hromić-Jahjefendić A. (2021). Meet the Insidious Players: Review of Viral Infections in Head and Neck Cancer Etiology with an Update on Clinical Trials. Microorganisms.

[39] de Martel C., Georges D., Bray F., Ferlay J., Clifford G.M. (2020). Global Burden of Cancer Attributable to Infections in 2018: A Worldwide Incidence Analysis. Lancet Glob. Health.

[40] Kellogg C., Kouznetsova V.L., Tsigelny I.F. (2021). Implications of Viral Infection in Cancer Development. Biochim. Biophys. Acta Rev. Cancer.

[41] Mesri E.A., Feitelson M.A., Munger K. (2014). Human Viral Oncogenesis: A Cancer Hallmarks Analysis. Cell Host Microbe.

[42] Akram N., Imran M., Noreen M., Ahmed F., Atif M., Fatima Z., Bilal Waqar A. (2017). Oncogenic Role of Tumor Viruses in Humans. Viral Immunol..

[43] Desfarges S., Ciuffi A., Witzany G. (2012). Viral Integration and Consequences on Host Gene Expression. Viruses: Essential Agents of Life.

[44] Wang Y., Wang H., Pan S., Hu T., Shen J., Zheng H., Xie S., Xie Y., Lu R., Guo L. (2018). Capable Infection of Hepatitis B Virus in Diffuse Large B-Cell Lymphoma. J. Cancer.

[45] Shibusawa M., Kidoguchi K., Tanimoto T., Gallamini A., Juweid M. (2021). Epstein-Barr Virus-Positive Diffuse Large B Cell Lymphoma. Lymphoma.

[46] Besson C., Lancar R., Prevot S., Algarte-Genin M., Delobel P., Bonnet F., Meyohas M.-C., Partisani M., Oberic L., Gabarre J. (2017). Outcomes for HIV-Associated Diffuse Large B-Cell Lymphoma in the Modern Combined Antiretroviral Therapy Era. AIDS.

[47] Visco C., Finotto S. (2014). Hepatitis C Virus and Diffuse Large B-Cell Lymphoma: Pathogenesis, Behavior and Treatment. World J. Gastroenterol..

[48] Beltran B.E., Quiñones P., Morales D., Revilla J.C., Alva J.C., Castillo J.J. (2012). Diffuse Large B-Cell Lymphoma in Human T-Lymphotropic Virus Type 1 Carriers. Leuk. Res. Treat..

[49] Amara K., Trimeche M., Ziadi S., Laatiri A., Hachana M., Sriha B., Mokni M., Korbi S. (2007). Presence of Simian Virus 40 DNA Sequences in Diffuse Large B-Cell Lymphomas in Tunisia Correlates with Aberrant Promoter Hypermethylation of Multiple Tumor Suppressor Genes. Int. J. Cancer.

[50] Amara K., Trimeche M., Ziadi S., Laatiri A., Mestiri S., Sriha B., Mokni M., Korbi S. (2008). Presence of Simian Virus 40 in Diffuse Large B-Cell Lymphomas in Tunisia Correlates with Germinal Center B-Cell Immunophenotype, t(14;18) Translocation, and P53 Accumulation. Mod. Pathol..

[51] Sreehari S. (2019). High Risk HPV, HSIL and Primary Diffuse Large B Cell Lymphoma of Cervix: An Unsual Case. IJCSMB.

[52] Van J., Dave A.A., Schwartz M., Mitchell J. (2020). S2443 A Case of HHV-8 Diffuse Large B-Cell Lymphoma—Not Otherwise Specified With Liver Infiltration. Am. J. Gastroenterol..

[53] Schiller J.T., Lowy D.R., Wu T.-C., Chang M.-H., Jeang K.-T. (2021). An Introduction to Virus Infections and Human Cancer. Viruses and Human Cancer.

[54] Montes-Moreno S., Odqvist L., Diaz-Perez J.A., Lopez A.B., de Villambrosía S.G., Mazorra F., Castillo M.E., Lopez M., Pajares R., García J.F. (2012). EBV-Positive Diffuse Large B-Cell Lymphoma of the Elderly Is an Aggressive Post-Germinal Center B-Cell Neoplasm Characterized by Prominent Nuclear Factor-KB Activation. Mod. Pathol..

[55] Mancao C., Hammerschmidt W. (2007). Epstein-Barr Virus Latent Membrane Protein 2A Is a B-Cell Receptor Mimic and Essential for B-Cell Survival. Blood.

[56] Mosialos G., Birkenbach M., Yalamanchili R., VanArsdale T., Ware C., Kieff E. (1995). The Epstein-Barr Virus Transforming Protein LMP1 Engages Signaling Proteins for the Tumor Necrosis Factor Receptor Family. Cell.

[57] Dawson C.W., Tramountanis G., Eliopoulos A.G., Young L.S. (2003). Epstein-Barr Virus Latent Membrane Protein 1 (LMP1) Activates the Phosphatidylinositol 3-Kinase/Akt Pathway to Promote Cell Survival and Induce Actin Filament Remodeling. J. Biol. Chem..

[58] Miura M., Naito T., Saito M. (2022). Current Perspectives in Human T-Cell Leukemia Virus Type 1 Infection and Its Associated Diseases. Front. Med..

[59] Panfil A.R., Martinez M.P., Ratner L., Green P.L. (2016). Human T-Cell Leukemia Virus-Associated Malignancy. Curr. Opin. Virol..

[60] Ahmadi Ghezeldasht S., Shirdel A., Assarehzadegan M.A., Hassannia T., Rahimi H., Miri R., Rezaee S.A.R. (2013). Human T Lymphotropic Virus Type I (HTLV-I) Oncogenesis: Molecular Aspects of Virus and Host Interactions in Pathogenesis of Adult T Cell Leukemia/Lymphoma (ATL). Iran J. Basic Med. Sci..

[61] Mota T.M., Jones R.B. (2019). HTLV-1 as a Model for Virus and Host Coordinated Immunoediting. Front. Immunol..

[62] Jeong S.-J., Pise-Masison C.A., Radonovich M.F., Park H.U., Brady J.N. (2005). Activated AKT Regulates NF-KappaB Activation, P53 Inhibition and Cell Survival in HTLV-1-Transformed Cells. Oncogene.

[63] Peloponese J.-M., Jeang K.-T. (2006). Role for Akt/Protein Kinase B and Activator Protein-1 in Cellular Proliferation Induced by the Human T-Cell Leukemia Virus Type 1 Tax Oncoprotein. J. Biol. Chem..

[64] Sun S.-C., Yamaoka S. (2005). Activation of NF-ΚB by HTLV-I and Implications for Cell Transformation. Oncogene.

[65] Iha H., Kibler K.V., Yedavalli V.R.K., Peloponese J.-M., Haller K., Miyazato A., Kasai T., Jeang K.-T. (2003). Segregation of NF-KappaB Activation through NEMO/IKKgamma by Tax and TNFalpha: Implications for Stimulus-Specific Interruption of Oncogenic Signaling. Oncogene.

[66] Grogg K.L., Miller R.F., Dogan A. (2007). HIV Infection and Lymphoma. J. Clin. Pathol..

[67] Bellan C., Lazzi S., De Falco G., Nyongo A., Giordano A., Leoncini L. (2003). Burkitt’s Lymphoma: New Insights into Molecular Pathogenesis. J. Clin. Pathol..

[68] Butel J.S., Lednicky J.A. (1999). Cell and Molecular Biology of Simian Virus 40: Implications for Human Infections and Disease. JNCI J. Natl. Cancer Inst..

[69] Sáenz-Robles M.T., Sullivan C.S., Pipas J.M. (2001). Transforming Functions of Simian Virus 40. Oncogene.

[70] Wang J.Y., Knudsen E.S., Welch P.J. (1994). The Retinoblastoma Tumor Suppressor Protein. Adv. Cancer Res..

[71] Ayee R., Ofori M.E., Wright E., Quaye O. (2020). Epstein Barr Virus Associated Lymphomas and Epithelia Cancers in Humans. J. Cancer.

[72] Nowalk A., Green M. (2016). Epstein-Barr Virus. Microbiol. Spectr..

[73] Bakkalci D., Jia Y., Winter J.R., Lewis J.E., Taylor G.S., Stagg H.R. (2020). Risk Factors for Epstein Barr Virus-Associated Cancers: A Systematic Review, Critical Appraisal, and Mapping of the Epidemiological Evidence. J. Glob. Health.

[74] Bauer M., Jasinski-Bergner S., Mandelboim O., Wickenhauser C., Seliger B. (2021). Epstein-Barr Virus-Associated Malignancies and Immune Escape: The Role of the Tumor Microenvironment and Tumor Cell Evasion Strategies. Cancers.

[75] Cohen J.I. (2000). Epstein-Barr Virus Infection. N. Engl. J. Med..

[76] Young L.S., Yap L.F., Murray P.G. (2016). Epstein-Barr Virus: More than 50 Years Old and Still Providing Surprises. Nat. Rev. Cancer.

[77] Santpere G., Darre F., Blanco S., Alcami A., Villoslada P., Mar Albà M., Navarro A. (2014). Genome-Wide Analysis of Wild-Type Epstein-Barr Virus Genomes Derived from Healthy Individuals of the 1000 Genomes Project. Genome Biol. Evol..

[78] Oyama T., Yamamoto K., Asano N., Oshiro A., Suzuki R., Kagami Y., Morishima Y., Takeuchi K., Izumo T., Mori S. (2007). Age-Related EBV-Associated B-Cell Lymphoproliferative Disorders Constitute a Distinct Clinicopathologic Group: A Study of 96 Patients. Clin. Cancer Res..

[79] Ok C.Y., Papathomas T.G., Medeiros L.J., Young K.H. (2013). EBV-Positive Diffuse Large B-Cell Lymphoma of the Elderly. Blood.

[80] Nicolae A., Pittaluga S., Abdullah S., Steinberg S.M., Pham T.A., Davies-Hill T., Xi L., Raffeld M., Jaffe E.S. (2015). EBV-Positive Large B-Cell Lymphomas in Young Patients: A Nodal Lymphoma with Evidence for a Tolerogenic Immune Environment. Blood.

[81] Hofscheier A., Ponciano A., Bonzheim I., Adam P., Lome-Maldonado C., Vela T., Cortes E., Ortiz-Hidalgo C., Fend F., Quintanilla-Martinez L. (2011). Geographic Variation in the Prevalence of Epstein-Barr Virus-Positive Diffuse Large B-Cell Lymphoma of the Elderly: A Comparative Analysis of a Mexican and a German Population. Mod. Pathol..

[82] Yoon H., Park S., Ju H., Ha S.Y., Sohn I., Jo J., Do I.-G., Min S., Kim S.J., Kim W.S. (2015). Integrated Copy Number and Gene Expression Profiling Analysis of Epstein-Barr Virus-Positive Diffuse Large B-Cell Lymphoma. Genes Chromosomes Cancer.

[83] Kato H., Karube K., Yamamoto K., Takizawa J., Tsuzuki S., Yatabe Y., Kanda T., Katayama M., Ozawa Y., Ishitsuka K. (2014). Gene Expression Profiling of Epstein-Barr Virus-Positive Diffuse Large B-Cell Lymphoma of the Elderly Reveals Alterations of Characteristic Oncogenetic Pathways. Cancer Sci..

[84] Kataoka K., Miyoshi H., Sakata S., Dobashi A., Couronné L., Kogure Y., Sato Y., Nishida K., Gion Y., Shiraishi Y. (2019). Frequent Structural Variations Involving Programmed Death Ligands in Epstein-Barr Virus-Associated Lymphomas. Leukemia.

[85] Gebauer N., Gebauer J., Hardel T.T., Bernard V., Biersack H., Lehnert H., Rades D., Feller A.C., Thorns C. (2015). Prevalence of Targetable Oncogenic Mutations and Genomic Alterations in Epstein-Barr Virus-Associated Diffuse Large B-Cell Lymphoma of the Elderly. Leuk. Lymphoma.

[86] Castillo J.J., Beltran B.E., Miranda R.N., Paydas S., Winer E.S., Butera J.N. (2011). Epstein-Barr Virus-Positive Diffuse Large B-Cell Lymphoma of the Elderly: What We Know so Far. Oncologist.

[87] Lu T.-X., Liang J.-H., Miao Y., Fan L., Wang L., Qu X.-Y., Cao L., Gong Q.-X., Wang Z., Zhang Z.-H. (2015). Epstein-Barr Virus Positive Diffuse Large B-Cell Lymphoma Predict Poor Outcome, Regardless of the Age. Sci. Rep..

[88] Murthy S.L., Hitchcock M.A., Endicott-Yazdani T.R., Watson J.T., Krause J.R. (2017). Epstein-Barr Virus-Positive Diffuse Large B-Cell Lymphoma. Bayl. Univ. Med. Cent. Proc..

[89] Bourbon E., Maucort-Boulch D., Fontaine J., Mauduit C., Sesques P., Safar V., Ferrant E., Golfier C., Ghergus D., Karlin L. (2021). Clinicopathological Features and Survival in EBV-Positive Diffuse Large B-Cell Lymphoma Not Otherwise Specified. Blood Adv..

[90] Beltran B.E., Castro D., Paredes S., Miranda R.N., Castillo J.J. (2020). EBV-Positive Diffuse Large B-Cell Lymphoma, Not Otherwise Specified: 2020 Update on Diagnosis, Risk-Stratification and Management. Am. J. Hematol..

[91] Long H.M., Taylor G.S., Rickinson A.B. (2011). Immune Defence against EBV and EBV-Associated Disease. Curr. Opin. Immunol..

[92] Rooney C.M., Smith C.A., Ng C.Y., Loftin S., Li C., Krance R.A., Brenner M.K., Heslop H.E. (1995). Use of Gene-Modified Virus-Specific T Lymphocytes to Control Epstein-Barr-Virus-Related Lymphoproliferation. Lancet.

[93] Savoldo B., Goss J.A., Hammer M.M., Zhang L., Lopez T., Gee A.P., Lin Y.-F., Quiros-Tejeira R.E., Reinke P., Schubert S. (2006). Treatment of Solid Organ Transplant Recipients with Autologous Epstein Barr Virus-Specific Cytotoxic T Lymphocytes (CTLs). Blood.

[94] Dong H., Strome S.E., Salomao D.R., Tamura H., Hirano F., Flies D.B., Roche P.C., Lu J., Zhu G., Tamada K. (2002). Tumor-Associated B7-H1 Promotes T-Cell Apoptosis: A Potential Mechanism of Immune Evasion. Nat. Med..

[95] Igakura T., Stinchcombe J.C., Goon P.K.C., Taylor G.P., Weber J.N., Griffiths G.M., Tanaka Y., Osame M., Bangham C.R.M. (2003). Spread of HTLV-I between Lymphocytes by Virus-Induced Polarization of the Cytoskeleton. Science.

[96] Manel N., Kim F.J., Kinet S., Taylor N., Sitbon M., Battini J.-L. (2003). The Ubiquitous Glucose Transporter GLUT-1 Is a Receptor for HTLV. Cell.

[97] Matsuoka M., Jeang K.-T. (2007). Human T-Cell Leukaemia Virus Type 1 (HTLV-1) Infectivity and Cellular Transformation. Nat. Rev. Cancer.

[98] Iwanaga R., Ohtani K., Hayashi T., Nakamura M. (2001). Molecular Mechanism of Cell Cycle Progression Induced by the Oncogene Product Tax of Human T-Cell Leukemia Virus Type I. Oncogene.

[99] Haller K., Wu Y., Derow E., Schmitt I., Jeang K.-T., Grassmann R. (2002). Physical Interaction of Human T-Cell Leukemia Virus Type 1 Tax with Cyclin-Dependent Kinase 4 Stimulates the Phosphorylation of Retinoblastoma Protein. Mol. Cell Biol..

[100] Cagigi A., Du L., Dang L.V.P., Grutzmeier S., Atlas A., Chiodi F., Pan-Hammarström Q., Nilsson A. (2009). CD27(-) B-Cells Produce Class Switched and Somatically Hyper-Mutated Antibodies during Chronic HIV-1 Infection. PLoS ONE.

[101] He B., Qiao X., Klasse P.J., Chiu A., Chadburn A., Knowles D.M., Moore J.P., Cerutti A. (2006). HIV-1 Envelope Triggers Polyclonal Ig Class Switch Recombination through a CD40-Independent Mechanism Involving BAFF and C-Type Lectin Receptors. J. Immunol..

[102] Ho J., Moir S., Kulik L., Malaspina A., Donoghue E.T., Miller N.J., Wang W., Chun T.-W., Fauci A.S., Holers V.M. (2007). Role for CD21 in the Establishment of an Extracellular HIV Reservoir in Lymphoid Tissues. J. Immunol..

[103] De Carvalho P.S., Leal F.E., Soares M.A. (2021). Clinical and Molecular Properties of Human Immunodeficiency Virus-Related Diffuse Large B-Cell Lymphoma. Front. Oncol..

[104] Grulich A.E., Li Y., McDonald A.M., Correll P.K., Law M.G., Kaldor J.M. (2001). Decreasing Rates of Kaposi’s Sarcoma and Non-Hodgkin’s Lymphoma in the Era of Potent Combination Anti-Retroviral Therapy. AIDS.

[105] Vandenhende M.-A., Roussillon C., Henard S., Morlat P., Oksenhendler E., Aumaitre H., Georget A., May T., Rosenthal E., Salmon D. (2015). Cancer-Related Causes of Death among HIV-Infected Patients in France in 2010: Evolution since 2000. PLoS ONE.

[106] Vilchez R.A., Butel J.S. (2003). SV40 in Human Brain Cancers and Non-Hodgkin’s Lymphoma. Oncogene.

[107] Stang E., Kartenbeck J., Parton R.G. (1997). Major Histocompatibility Complex Class I Molecules Mediate Association of SV40 with Caveolae. MBoC.

[108] Toscano M.G., de Haan P. (2018). How Simian Virus 40 Hijacks the Intracellular Protein Trafficking Pathway to Its Own Benefit … and Ours. Front. Immunol..

[109] Rotondo J.C., Mazzoni E., Bononi I., Tognon M., Martini F. (2019). Association Between Simian Virus 40 and Human Tumors. Front. Oncol..

[110] Sullivan C.S., Pipas J.M. (2002). T Antigens of Simian Virus 40: Molecular Chaperones for Viral Replication and Tumorigenesis. Microbiol. Mol. Biol. Rev..

[111] Bocchetta M., Eliasz S., De Marco M.A., Rudzinski J., Zhang L., Carbone M. (2008). The SV40 Large T Antigen-P53 Complexes Bind and Activate the Insulin-like Growth Factor-I Promoter Stimulating Cell Growth. Cancer Res..

[112] Atkin S.J.L., Griffin B.E., Dilworth S.M. (2009). Polyoma Virus and Simian Virus 40 as Cancer Models: History and Perspectives. Semin. Cancer Biol..

[113] Tsai K.-N., Kuo C.-F., Ou J.-H.J. (2018). Mechanisms of Hepatitis B Virus Persistence. Trends Microbiol..

[114] Tong S., Revill P. (2016). Overview of Hepatitis B Viral Replication and Genetic Variability. J. Hepatol..

[115] Herrscher C., Roingeard P., Blanchard E. (2020). Hepatitis B Virus Entry into Cells. Cells.

[116] Jiang B., Hildt E. (2020). Intracellular Trafficking of HBV Particles. Cells.

[117] Vincent I.E., Zannetti C., Lucifora J., Norder H., Protzer U., Hainaut P., Zoulim F., Tommasino M., Trépo C., Hasan U. (2011). Hepatitis B Virus Impairs TLR9 Expression and Function in Plasmacytoid Dendritic Cells. PLoS ONE.

[118] Tsutsumi Y., Yamamoto Y., Ito S., Ohigashi H., Shiratori S., Naruse H., Teshima T. (2015). Hepatitis B Virus Reactivation with a Rituximab-Containing Regimen. World J. Hepatol..

[119] Douam F., Lavillette D., Cosset F.-L. (2015). The Mechanism of HCV Entry into Host Cells. Progress in Molecular Biology and Translational Science.

[120] Burlone M.E., Budkowska A. (2009). Hepatitis C Virus Cell Entry: Role of Lipoproteins and Cellular Receptors. J. Gen. Virol..

[121] Machida K., Cheng K.T.-N., Sung V.M.-H., Shimodaira S., Lindsay K.L., Levine A.M., Lai M.-Y., Lai M.M.C. (2004). Hepatitis C Virus Induces a Mutator Phenotype: Enhanced Mutations of Immunoglobulin and Protooncogenes. Proc. Natl. Acad. Sci. USA.

[122] Couronné L., Bachy E., Roulland S., Nadel B., Davi F., Armand M., Canioni D., Michot J.M., Visco C., Arcaini L. (2018). From Hepatitis C Virus Infection to B-Cell Lymphoma. Ann. Oncol..

[123] Machida K., Cheng K.T.-H., Pavio N., Sung V.M.-H., Lai M.M.C. (2005). Hepatitis C Virus E2-CD81 Interaction Induces Hypermutation of the Immunoglobulin Gene in B Cells. J. Virol..

[124] Ishikawa T., Shibuya K., Yasui K., Mitamura K., Ueda S. (2003). Expression of Hepatitis C Virus Core Protein Associated with Malignant Lymphoma in Transgenic Mice. Comp. Immunol. Microbiol. Infect. Dis..

[125] Dai B., Chen A.Y., Corkum C.P., Peroutka R.J., Landon A., Houng S., Muniandy P.A., Zhang Y., Lehrmann E., Mazan-Mamczarz K. (2016). Hepatitis C Virus Upregulates B-Cell Receptor Signaling: A Novel Mechanism for HCV-Associated B-Cell Lymphoproliferative Disorders. Oncogene.

[126] Machida K., Cheng K.T.-H., Sung V.M.-H., Lee K.J., Levine A.M., Lai M.M.C. (2004). Hepatitis C Virus Infection Activates the Immunologic (Type II) Isoform of Nitric Oxide Synthase and Thereby Enhances DNA Damage and Mutations of Cellular Genes. J. Virol..

[127] Hosry J., Mahale P., Turturro F., Miranda R.N., Economides M.P., Granwehr B.P., Torres H.A. (2016). Antiviral Therapy Improves Overall Survival in Hepatitis C Virus-Infected Patients Who Develop Diffuse Large B-Cell Lymphoma. Int. J. Cancer.

[128] Michot J.-M., Canioni D., Driss H., Alric L., Cacoub P., Suarez F., Sibon D., Thieblemont C., Dupuis J., Terrier B. (2015). Antiviral Therapy Is Associated with a Better Survival in Patients with Hepatitis C Virus and B-Cell Non-Hodgkin Lymphomas, ANRS HC-13 Lympho-C Study. Am. J. Hematol..

[129] Valcarcel B., Ampuero G.S., de la Cruz-Ku G., Enriquez D.J., Malpica L. (2022). Outcomes of HTLV-1 Carriers with Diffuse Large B-Cell Lymphoma: A Single-Center Retrospective Matched Cohort Study. Clin. Lymphoma. Myeloma. Leuk..

[130] Kasinathan G., Sathar J. (2022). Peripheral Lymphocytosis Presenting as EBV/HTLV-1 Co-Infection Adult T-Cell Leukemia. Hematol. Transfus. Cell Ther..

[131] Proietti F.A., Carneiro-Proietti A.B.F., Catalan-Soares B.C., Murphy E.L. (2005). Global Epidemiology of HTLV-I Infection and Associated Diseases. Oncogene.

[132] Gonçalves D.U., Proietti F.A., Ribas J.G.R., Araújo M.G., Pinheiro S.R., Guedes A.C., Carneiro-Proietti A.B.F. (2010). Epidemiology, Treatment, and Prevention of Human T-Cell Leukemia Virus Type 1-Associated Diseases. Clin. Microbiol. Rev..

[133] Zhao T. (2016). The Role of HBZ in HTLV-1-Induced Oncogenesis. Viruses.

[134] Zhao T., Matsuoka M. (2012). HBZ and Its Roles in HTLV-1 Oncogenesis. Front. Microbiol..

[135] Suefuji H., Ohshima K., Hayabuchi N., Nakamura K., Kikuchi M. (2003). HTLV-1 Carriers with B-Cell Lymphoma of Localized Stage Head and Neck: Prognosis, Clinical and Immunopathological Features. Br. J. Haematol..

[136] Valcarcel B., Enriquez D.J., Sandival-Ampuero G., Aviles-Perez U., Haro J.C., Alcarraz C., Quintana S., Villena M., Dueñas D., Casavilca S. (2020). Clinical Characteristics and Outcome of Diffuse Large B Cell Lymphoma Among HTLV-1 Carriers in Peru: A Matched Cohort Study. Blood.

[137] Isaguliants M., Bayurova E., Avdoshina D., Kondrashova A., Chiodi F., Palefsky J.M. (2021). Oncogenic Effects of HIV-1 Proteins, Mechanisms Behind. Cancers.

[138] Laurence J., Astrin S.M. (1991). Human Immunodeficiency Virus Induction of Malignant Transformation in Human B Lymphocytes. Proc. Natl. Acad. Sci. USA.

[139] Planès R., Serrero M., Leghmari K., BenMohamed L., Bahraoui E. (2018). HIV-1 Envelope Glycoproteins Induce the Production of TNF-α and IL-10 in Human Monocytes by Activating Calcium Pathway. Sci. Rep..

[140] Malaspina A., Moir S., Kottilil S., Hallahan C.W., Ehler L.A., Liu S., Planta M.A., Chun T.-W., Fauci A.S. (2003). Deleterious Effect of HIV-1 Plasma Viremia on B Cell Costimulatory Function. J. Immunol..

[141] Yan J., Zhang S., Sun J., Xu J., Zhang X. (2017). Irreversible Phenotypic Perturbation and Functional Impairment of B Cells during HIV-1 Infection. Front. Med..

[142] Wu J., Miao Y., Qian C., Tao P., Wang X., Dong X., Li X., Lou J., Liang J., Xu W. (2021). Clinical Characteristics and Outcomes in HIV-Associated Diffuse Large B-Cell Lymphoma in China: A Retrospective Single-Center Study. J. Cancer.

[143] Diamond C., Taylor T.H., Aboumrad T., Anton-Culver H. (2006). Changes in Acquired Immunodeficiency Syndrome-Related Non-Hodgkin Lymphoma in the Era of Highly Active Antiretroviral Therapy: Incidence, Presentation, Treatment, and Survival. Cancer.

[144] Hoffmann C., Wolf E., Fätkenheuer G., Buhk T., Stoehr A., Plettenberg A., Stellbrink H.-J., Jaeger H., Siebert U., Horst H.-A. (2003). Response to Highly Active Antiretroviral Therapy Strongly Predicts Outcome in Patients with AIDS-Related Lymphoma. AIDS.

[145] Tsuyama N., Sakata S., Baba S., Mishima Y., Nishimura N., Ueda K., Yokoyama M., Terui Y., Hatake K., Kitagawa M. (2017). BCL2 Expression in DLBCL: Reappraisal of Immunohistochemistry with New Criteria for Therapeutic Biomarker Evaluation. Blood.

[146] Philippe L., Lancar R., Laurent C., Algarte-Genin M., Chassagne-Clément C., Fabiani B., Pierre Chenard M., Lazure T., Parrens M., Charlotte F. (2020). In Situ BCL2 Expression Is an Independent Prognostic Factor in HIV-Associated DLBCL, a LYMPHOVIR Cohort Study. Br. J. Haematol..

[147] Stratton K., Almario D.A., McCormick M.C. (2002). Immunization Safety Review: SV40 Contamination of Polio Vaccine and Cancer.

[148] McNees A.L., Butel J.S. (2008). Simian Virus 40. Encyclopedia of Virology.

[149] Tognon M., Luppi M., Corallini A., Taronna A., Barozzi P., Rotondo J.C., Comar M., Casali M.V., Bovenzi M., D’Agostino A. (2015). Immunologic Evidence of a Strong Association between Non-Hodgkin Lymphoma and Simian Virus 40: NHL and SV40. Cancer.

[150] Jasani B., Cristaudo A., Emri S.A., Gazdar A.F., Gibbs A., Krynska B., Miller C., Mutti L., Radu C., Tognon M. (2001). Association of SV40 with Human Tumours. Semin. Cancer Biol..

[151] Heinsohn S., Scholz R., Kabisch H. (2011). SV40 and P53 as Team Players in Childhood Lymphoproliferative Disorders. Int. J. Oncol..

[152] Pipas J.M., Levine A.J. (2001). Role of T Antigen Interactions with P53 in Tumorigenesis. Semin. Cancer Biol..

[153] Vilchez R.A., Madden C.R., Kozinetz C.A., Halvorson S.J., White Z.S., Jorgensen J.L., Finch C.J., Butel J.S. (2002). Association between Simian Virus 40 and Non-Hodgkin Lymphoma. Lancet.

[154] Zekri A.-R., Mohamed W., Bahnassy A., Refat L., Khaled M., Shalaby S., Hafez M. (2007). Detection of Simian Virus 40 DNA Sequences in Egyptian Patients with Different Hematological Malignancies. Leuk. Lymphoma..

[155] MacKenzie J., Wilson K.S., Perry J., Gallagher A., Jarrett R.F. (2003). Association Between Simian Virus 40 DNA and Lymphoma in the United Kingdom. JNCI J. Natl. Cancer Inst..

[156] Rollison D.E., Helzlsouer K.J., Halsey N.A., Shah K.V., Viscidi R.P. (2005). Markers of Past Infection with Simian Virus 40 (SV40) and Risk of Incident Non-Hodgkin Lymphoma in a Maryland Cohort. Cancer Epidemiol. Biomark. Prev..

[157] Dinantia N., Anggorowati N. (2021). Expression of Simian Virus 40 Large T-Antigen: A Case Control Study of Non-Hodgkin Lymphoma. Asian Pac. J. Cancer Biol..

[158] Lamontagne R.J., Bagga S., Bouchard M.J. (2016). Hepatitis B Virus Molecular Biology and Pathogenesis. Hepatoma Res..

[159] Schulze A., Gripon P., Urban S. (2007). Hepatitis B Virus Infection Initiates with a Large Surface Protein-Dependent Binding to Heparan Sulfate Proteoglycans. Hepatology.

[160] Iwamoto M., Saso W., Sugiyama R., Ishii K., Ohki M., Nagamori S., Suzuki R., Aizaki H., Ryo A., Yun J.-H. (2019). Epidermal Growth Factor Receptor Is a Host-Entry Cofactor Triggering Hepatitis B Virus Internalization. Proc. Natl. Acad. Sci. USA.

[161] Trebicka J., Bork P., Krag A., Arumugam M. (2021). Utilizing the Gut Microbiome in Decompensated Cirrhosis and Acute-on-Chronic Liver Failure. Nat. Rev. Gastroenterol. Hepatol..

[162] Ren W., Ye X., Su H., Li W., Liu D., Pirmoradian M., Wang X., Zhang B., Zhang Q., Chen L. (2018). Genetic Landscape of Hepatitis B Virus–Associated Diffuse Large B-Cell Lymphoma. Blood.

[163] Wieland S., Thimme R., Purcell R.H., Chisari F.V. (2004). Genomic Analysis of the Host Response to Hepatitis B Virus Infection. Proc. Natl. Acad. Sci. USA.

[164] Luangsay S., Gruffaz M., Isorce N., Testoni B., Michelet M., Faure-Dupuy S., Maadadi S., Ait-Goughoulte M., Parent R., Rivoire M. (2015). Early Inhibition of Hepatocyte Innate Responses by Hepatitis B Virus. J. Hepatol..

[165] Evens A.M., Jovanovic B.D., Su Y.-C., Raisch D.W., Ganger D., Belknap S.M., Dai M.-S., Chiu B.-C.C., Fintel B., Cheng Y. (2011). Rituximab-Associated Hepatitis B Virus (HBV) Reactivation in Lymphoproliferative Diseases: Meta-Analysis and Examination of FDA Safety Reports. Ann. Oncol..

[166] Cabrerizo M., Bartolomé J., Caramelo C., Barril G., Carreno V. (2000). Molecular Analysis of Hepatitis B Virus DNA in Serum and Peripheral Blood Mononuclear Cells from Hepatitis B Surface Antigen-Negative Cases. Hepatology.

[167] Loggi E., Gamal N., Bihl F., Bernardi M., Andreone P. (2014). Adaptive Response in Hepatitis B Virus Infection. J. Viral. Hepat..

[168] Deng L., Song Y., Young K.H., Hu S., Ding N., Song W., Li X., Shi Y., Huang H., Liu W. (2015). Hepatitis B Virus-Associated Diffuse Large B-Cell Lymphoma: Unique Clinical Features, Poor Outcome, and Hepatitis B Surface Antigen-Driven Origin. Oncotarget.

[169] Zhang M.-Y., Gao F., Zhao Y.-W., Ni B.-W., Huang H.-H., Hou J. (2022). Inferior Survival and Frequent Hepatic Dysfunction in Non-Hodgkin’s Lymphoma Patients with HBV Infection: A Systematic Review and Meta-Analysis. Hematology.

[170] Dalia S., Chavez J., Castillo J.J., Sokol L. (2013). Hepatitis B Infection Increases the Risk of Non-Hodgkin Lymphoma: A Meta-Analysis of Observational Studies. Leuk. Res..

[171] Marcucci G., Perrotti D., Caligiuri M.A. (2003). Understanding the Molecular Basis of Imatinib Mesylate Therapy in Chronic Myelogenous Leukemia and the Related Mechanisms of Resistance: Commentary Re: A. N. Mohamed et al., The Effect of Imatinib Mesylate on Patients with Philadelphia Chromosome-Positive Chronic Myeloid Leukemia with Secondary Chromosomal Aberrations. Clin. Cancer Res., 9: 1333–1337, 2003. Clin. Cancer Res..

[172] Yan X., Zhou M., Lou Z., Mu Q., Sheng L., Zhang P., Wang Y., Ouyang G. (2018). Diffuse Large B-Cell Lymphoma with Concurrent Hepatitis B Virus Infection in the MabThera Era: Unique Clinical Features and Worse Outcomes. J. Cancer Res. Ther..

[173] Pfreundschuh M., Ho A.D., Cavallin-Stahl E., Wolf M., Pettengell R., Vasova I., Belch A., Walewski J., Zinzani P.-L., Mingrone W. (2008). Prognostic Significance of Maximum Tumour (Bulk) Diameter in Young Patients with Good-Prognosis Diffuse Large-B-Cell Lymphoma Treated with CHOP-like Chemotherapy with or without Rituximab: An Exploratory Analysis of the MabThera International Trial Group (MInT) Study. Lancet Oncol..

[174] Mozessohn L., Chan K.K.W., Feld J.J., Hicks L.K. (2015). Hepatitis B Reactivation in HBsAg-Negative/HBcAb-Positive Patients Receiving Rituximab for Lymphoma: A Meta-Analysis. J. Viral. Hepat..

[175] Penin F., Dubuisson J., Rey F.A., Moradpour D., Pawlotsky J.-M. (2004). Structural Biology of Hepatitis C Virus. Hepatology.

[176] Moradpour D., Penin F., Bartenschlager R. (2013). Hepatitis C Virus Proteins: From Structure to Function. Hepatitis C Virus: From Molecular Virology to Antiviral Therapy.

[177] Spera A.M. (2022). Safety of Direct Acting Antiviral Treatment for Hepatitis C in Oncologic Setting: A Clinical Experience and a Literature Review. World J. Hepatol..

[178] Sène D., Limal N., Ghillani-Dalbin P., Saadoun D., Piette J.-C., Cacoub P. (2007). Hepatitis C Virus-Associated B-Cell Proliferation--the Role of Serum B Lymphocyte Stimulator (BLyS/BAFF). Rheumatology.

[179] Giordano T.P., Henderson L., Landgren O., Chiao E.Y., Kramer J.R., El-Serag H., Engels E.A. (2007). Risk of Non-Hodgkin Lymphoma and Lymphoproliferative Precursor Diseases in US Veterans With Hepatitis C Virus. JAMA.

[180] Fiorino S. (2015). Possible Association between Hepatitis C Virus and Malignancies Different from Hepatocellular Carcinoma: A Systematic Review. World J. Gastroenterol..

[181] Nieters A., Kallinowski B., Brennan P., Ott M., Maynadié M., Benavente Y., Foretova L., Cocco P.L., Staines A., Vornanen M. (2006). Hepatitis C and Risk of Lymphoma: Results of the European Multicenter Case-Control Study EPILYMPH. Gastroenterology.

[182] De Sanjose S., Benavente Y., Vajdic C.M., Engels E.A., Morton L.M., Bracci P.M., Spinelli J.J., Zheng T., Zhang Y., Franceschi S. (2008). Hepatitis C and Non-Hodgkin Lymphoma Among 4784 Cases and 6269 Controls From the International Lymphoma Epidemiology Consortium. Clin. Gastroenterol. Hepatol..

[183] Pozzato G., Mazzaro C., Gattei V. (2017). Hepatitis C Virus–Associated Non-Hodgkin Lymphomas. Clin. Liver Dis..

[184] Pioltelli P., Zehender G., Monti G., Monteverde A., Galli M. (1996). HCV and Non-Hodgkin Lymphoma. Lancet.

[185] Mele A., Pulsoni A., Bianco E., Musto P., Szklo A., Sanpaolo M.G., Iannitto E., De Renzo A., Martino B., Liso V. (2003). Hepatitis C Virus and B-Cell Non-Hodgkin Lymphomas: An Italian Multicenter Case-Control Study. Blood.

[186] Zuckerman T., Rowe J. (2014). Pathogenesis and Prognostication in Acute Lymphoblastic Leukemia. F1000Prime Rep..

[187] Zuckerman E., Zuckerman T., Sahar D., Streichman S., Attias D., Sabo E., Yeshurun D., Rowe J.M. (2001). The Effect of Antiviral Therapy on t(14;18) Translocation and Immunoglobulin Gene Rearrangement in Patients with Chronic Hepatitis C Virus Infection. Blood.

[188] Giannelli F., Moscarella S., Giannini C., Caini P., Monti M., Gragnani L., Romanelli R.G., Solazzo V., Laffi G., La Villa G. (2003). Effect of Antiviral Treatment in Patients with Chronic HCV Infection and t(14;18) Translocation. Blood.

[189] Ferri C., Caracciolo F., Zignego A.L., Civita L.L., Monti M., Longombardo G., Lombardini F., Greco F., Capochiani E., Mazzoni A. (1994). Hepatitis C Virus Infection in Patients with Non-Hodgkin’s Lymphoma. Br. J. Haematol..

[190] Galati G., Rampa L., Vespasiani-Gentilucci U., Marino M., Pisani F., Cota C., Guidi A., Picardi A. (2016). Hepatitis C and Double-Hit B Cell Lymphoma Successfully Treated by Antiviral Therapy. World J. Hepatol..

[191] Duberg A.-S., Nordström M., Törner A., Reichard O., Strauss R., Janzon R., Bäck E., Ekdahl K. (2005). Non-Hodgkin’s Lymphoma and Other Nonhepatic Malignancies in Swedish Patients with Hepatitis C Virus Infection. Hepatology.

[192] Luppi M., Longo G., Ferrari M.G., Barozzi P., Marasca R., Morselli M., Valenti C., Mascia T., Vandelli L., Vallisa D. (1998). Clinico-Pathological Characterization of Hepatitis C Virus-Related B-Cell Non-Hodgkin’s Lymphomas without Symptomatic Cryoglobulinemia. Ann. Oncol..

[193] Visco C., Arcaini L., Brusamolino E., Burcheri S., Ambrosetti A., Merli M., Bonoldi E., Chilosi M., Viglio A., Lazzarino M. (2006). Distinctive Natural History in Hepatitis C Virus Positive Diffuse Large B-Cell Lymphoma: Analysis of 156 Patients from Northern Italy. Ann. Oncol..

[194] Besson C., Canioni D., Lepage E., Pol S., Morel P., Lederlin P., Van Hoof A., Tilly H., Gaulard P., Coiffier B. (2006). Characteristics and Outcome of Diffuse Large B-Cell Lymphoma in Hepatitis C Virus-Positive Patients in LNH 93 and LNH 98 Groupe d’Etude Des Lymphomes de l’Adulte Programs. J. Clin. Oncol..

[195] De Renzo A., Perna F., Persico M., Notaro R., Mainolfi C., de Sio I., Ciancia G., Picardi M., Del Vecchio L., Pane F. (2008). Excellent Prognosis and Prevalence of HCV Infection of Primary Hepatic and Splenic Non-Hodgkin’s Lymphoma. Eur. J. Haematol..

[196] Chen Z., Zhu Y., Ren Y., Tong Y., Hua X., Zhu F., Huang L., Liu Y., Luo Y., Lu W. (2011). Hepatitis C Virus Protects Human B Lymphocytes from Fas-Mediated Apoptosis via E2-CD81 Engagement. PLoS ONE.

[197] Stamataki Z., Shannon-Lowe C., Shaw J., Mutimer D., Rickinson A.B., Gordon J., Adams D.H., Balfe P., McKeating J.A. (2009). Hepatitis C Virus Association with Peripheral Blood B Lymphocytes Potentiates Viral Infection of Liver-Derived Hepatoma Cells. Blood.

[198] Peveling-Oberhag J., Arcaini L., Hansmann M.-L., Zeuzem S. (2013). Hepatitis C-Associated B-Cell Non-Hodgkin Lymphomas. Epidemiology, Molecular Signature and Clinical Management. J. Hepatol..

[199] Bachy E., Besson C., Suarez F., Hermine O. (2010). Hepatitis C Virus Infection and Lymphoma. Mediterr. J. Hematol. Infect. Dis..

[200] Merli M., Visco C., Spina M., Luminari S., Ferretti V.V., Gotti M., Rattotti S., Fiaccadori V., Rusconi C., Targhetta C. (2014). Outcome Prediction of Diffuse Large B-Cell Lymphomas Associated with Hepatitis C Virus Infection: A Study on Behalf of the Fondazione Italiana Linfomi. Haematologica.

[201] Xuan C., Steward K.K., Timmerman J.M., Morrison S.L. (2010). Targeted Delivery of Interferon-Alpha via Fusion to Anti-CD20 Results in Potent Antitumor Activity against B-Cell Lymphoma. Blood.

[202] Zheng Z.-M., Baker C.C. (2006). Papillomavirus Genome Structure, Expression, and Post-Transcriptional Regulation. Front. Biosci..

[203] Ren X., Cheng Y., Wu S., Zeng X., Shi X., Ling Q., Li Z., Liang Z., Wang B. (2020). Primary Non-Hodgkin Lymphoma of the Tongue Base: The Clinicopathology of Seven Cases and Evaluation of HPV and EBV Status. Diagn. Pathol..

[204] Heawchaiyaphum C., Ekalaksananan T., Patarapadungkit N., Vatanasapt P., Pientong C. (2022). Association of Human Papillomavirus and Epstein-Barr Virus Infection with Tonsil Cancer in Northeastern Thailand. Asian Pac. J. Cancer Prev..

[205] Gillson M.L., Richard A.F. (1997). Human Herpesvirus-8. Curr. Opin. Oncol..

[206] Zhao Y., Maule J., Li Y., Neff J., McCall C.M., Hao T., Yang W., Rehder C., Yang L.-H., Wang E. (2019). Sequential Development of Human Herpes Virus 8-Positive Diffuse Large B-Cell Lymphoma and Chronic Myelomonocytic Leukemia in a 59 Year Old Female Patient with Hemoglobin SC Disease. Pathol. Res. Pract..

[207] Tanière P., Manai A., Charpentier R., Terdjman P., Boucheron S., Cordier J.F., Berger F. (1998). Pyothorax-Associated Lymphoma: Relationship with Epstein-Barr Virus, Human Herpes Virus-8 and Body Cavity-Based High Grade Lymphomas. Eur. Respir. J..

